# Sequential use of midazolam and dexmedetomidine for long-term sedation may reduce weaning time in selected critically ill, mechanically ventilated patients: a randomized controlled study

**DOI:** 10.1186/s13054-022-03967-5

**Published:** 2022-05-03

**Authors:** Yongfang Zhou, Jie Yang, Bo Wang, Peng Wang, Zhen Wang, Yunqin Yang, Guopeng Liang, Xiaorong jing, Xiaodong Jin, Zhongwei Zhang, Yiyun Deng, Chenggong Hu, Xuelian Liao, Wanhong Yin, Zhihong Tang, Yongming Tian, Liyuan Tao, Yan Kang

**Affiliations:** 1 Department of Critical Care Medicine, West China Hospital of Sichuan University, Guoxue Alley 37#, Wuhou District, Chengdu, 610041 Sichuan China; 2 Department of Respiratory and Critical Care Medicine, West China Hospital of Sichuan University, Chengdu, 610041 Sichuan China; 3 Research Center of Clinical Epidemiology, Peking University Third Hospital, Beijing, 100191 China

**Keywords:** Midazolam, Propofol, Dexmedetomidine, Sequential sedation, Critically ill, Mechanical ventilation

## Abstract

**Background:**

Current sedatives have different side effects in long-term sedation. The sequential use of midazolam and dexmedetomidine for prolonged sedation may have distinct advantages. We aimed to evaluate the efficacy and safety of the sequential use of midazolam and either dexmedetomidine or propofol, and the use of midazolam alone in selected critically ill, mechanically ventilated patients.

**Methods:**

This single-center, randomized controlled study was conducted in medical and surgical ICUs in a tertiary, academic medical center. Patients enrolled in this study were critically ill, mechanically ventilated adult patients receiving midazolam, with anticipated mechanical ventilation for ≥ 72 h. They passed the spontaneous breathing trial (SBT) safety screen, underwent a 30-min-SBT without indication for extubation and continued to require sedation. Patients were randomized into group M-D (midazolam was switched to dexmedetomidine), group M-P (midazolam was switched to propofol), and group M (sedation with midazolam alone), and sedatives were titrated to achieve the targeted sedation range (RASS − 2 to 0).

**Results:**

Total 252 patients were enrolled. Patients in group M-D had an earlier recovery, faster extubation, and more percentage of time at the target sedation level than those in group M-P and group M (all *P* < 0.001). They also experienced less weaning time (25.0 h vs. 49.0 h; HR1.47, 95% CI 1.05 to 2.06; *P* = 0.025), and a lower incidence of delirium (19.5% vs. 43.8%, *P* = 0.002) than patients in group M. Recovery (*P* < 0.001), extubation (*P* < 0.001), and weaning time (*P* = 0.048) in group M-P were shorter than in group M, while the acquisition cost of sedative drug was more expensive than other groups (both *P* < 0.001). There was no significant difference in adverse events among these groups (all *P* > 0.05).

**Conclusions:**

The sequential use of midazolam and dexmedetomidine was an effective and safe sedation strategy for long-term sedation and could provide clinically relevant benefits for selected critically ill, mechanically ventilated patients.

***Trial registration*:**

NCT02528513. Registered August 19, 2015.

**Supplementary Information:**

The online version contains supplementary material available at 10.1186/s13054-022-03967-5.

## Background

Sedation is an essential component of care in critically ill, mechanically ventilated patients, which should ideally control anxiety and agitation and provide amnesia while minimizing adverse effects [1–3]. Current sedatives have different side-effect profiles and remain problematic in long-term sedation [4]. Midazolam and propofol used to be the first-line agents for sedation in mechanically ventilated patients [5–7]. Midazolam is a potent anxiolytic, hypnotic, and sedative with a drawback of unpredictable accumulation of its active metabolite, and midazolam can induce anterograde amnesia. Nevertheless, previous studies showed that midazolam used for long-term sedation in mechanically ventilated patients was associated with worse outcomes, including delayed recovery, prolonged mechanical ventilation, and possible development of delirium [4, 8–12]. Propofol, a sedative-hypnotic agent, was associated with a dose-dependent effect and faster recovery without accumulation [4, 8, 9, 11, 12]. However, high dose or prolonged use of propofol may cause hypertriglyceridemia, uncommon fatal propofol infusion syndrome, respiratory drive depression, and hypotension because of systemic vasodilation [4, 9, 11, 14, 15].

Unlike other sedatives, dexmedetomidine—a highly selective central alpha-2 adrenergic agonist with both analgesic and sedative effects, notable for its ability to provide light sedation, analgesia, and physiologic-like sleep, as well as its minimal effect on respiratory drive—has been shown to result in a more awake and interactive patient, a lower incidence of delirium, fewer days on ventilator, and an earlier ICU discharge [4, 16–27]. However, previous studies reported dexmedetomidine was more applicable for light to moderate sedation than deep sedation despite the use of the maximum dose of dexmedetomidine (1.4 µg/kg/h) [19, 20, 23, 28] and was associated with markedly increased fentanyl needs in patients with RASS target − 3 or deeper [21]; what’s more, bradycardia and hypotension were more common with dexmedetomidine [22–24, 26, 28, 29].

Although the current guideline recommends using light sedation with nonbenzodiazepine sedatives [2, 30], in real clinical practice, midazolam is still frequently used, especially when there is a need for deep sedation such as severe respiratory failure, patient-ventilator asynchrony despite optimizing ventilator settings, compromised hemodynamics, and in the setting of resource-limited countries [5–7, 28, 31, 32]. By considering the advantages and disadvantages of different sedative drugs, clinicians should optimize sedation strategies according to sedatives’ pharmacological properties and patient’s characteristics and sedation requirements. Our previous study showed that the sequential use of midazolam and propofol according to the ventilator weaning process was associated with faster recovery and extubation, lower ICU treatment cost, and a decreased incidence of agitation than midazolam alone; as well as lower occurrence of hypotension and pharmaceutic cost than propofol alone [33]. There is still a lack of research regarding the sequential use of midazolam and dexmedetomidine. It was hypothesized that the sequential use of midazolam and dexmedetomidine based on the weaning process could improve outcomes. To test our hypothesis, we assessed the safety and efficacy of the sequential use of midazolam and either dexmedetomidine or propofol, or midazolam used alone for long-term sedation in critically ill, mechanically ventilated patients receiving midazolam as determined by treating physician.

## Methods

### Study design

This single-center, randomized, open-label, controlled trial was performed at West China Medical Center, Sichuan University, Chengdu, China, between December 2015 and June 2018. The Ethics Committee of West China Hospital of Sichuan University approved this trial (the approval number #2015-107). Written informed consent was obtained from legally authorized representatives.

### Patients

Patients were enrolled through two phases: a screening phase and a confirmatory phase.

In the screening phase, all intubated, mechanically ventilated patients were screened for initial inclusion and exclusion criteria within 12 h of admission to the ICU. The initial inclusion criteria were age between 18 and 80 years, with an anticipated mechanical ventilation duration of ≥ 72 h, and patients receiving fentanyl for analgesia and midazolam for sedation as determined by the treating physician. The exclusion criteria are listed in S4.2.1, Additional file 1. Once the patients passed the screening phase, study personnel would follow up with them daily. Patients continued to receive fentanyl for analgesia and midazolam for sedation, with titration to achieve the target analgesia level [34] and the individual sedation goal (details listed in S4.2.1, Additional file 1). All the patients were managed with a daily spontaneous awakening trial (SAT) followed by a spontaneous breathing trial (SBT) throughout the mechanical ventilation period [35].

During the confirmatory phase, clinicians managed patients with the spontaneous awakening trial (SAT) from the next morning after screening. Once patients passed the SAT, respiratory therapists assessed patients using spontaneous breath trial (SBT) safety screen [35] (details in S4.2.2, Additional file 1). Patients who passed the SBT safety screen underwent a 30-min SBT trial with pressure support 5–8 cmH_2_O, positive end-expiratory pressure 5 cmH_2_O, and a fraction of inspired oxygen 40% [36]. Patients failed an SBT trial when they showed any of the following failure signs: respiratory rate > 35 breaths/min or < 8 breaths/min, hypoxemia (SPO_2_ or SaO_2_ < 90%), abrupt changes in mental status, unstable cardiovascular status with heart rate and blood pressure changing more than 20% from the previous level, acute cardiac arrhythmia, tachycardia (> 140 beats/min) or bradycardia (< 60 beats/min), shortness of breath, or signs of increased work of breath such as the use of accessory muscles or abdominal paradox.

**Fig. 1:**
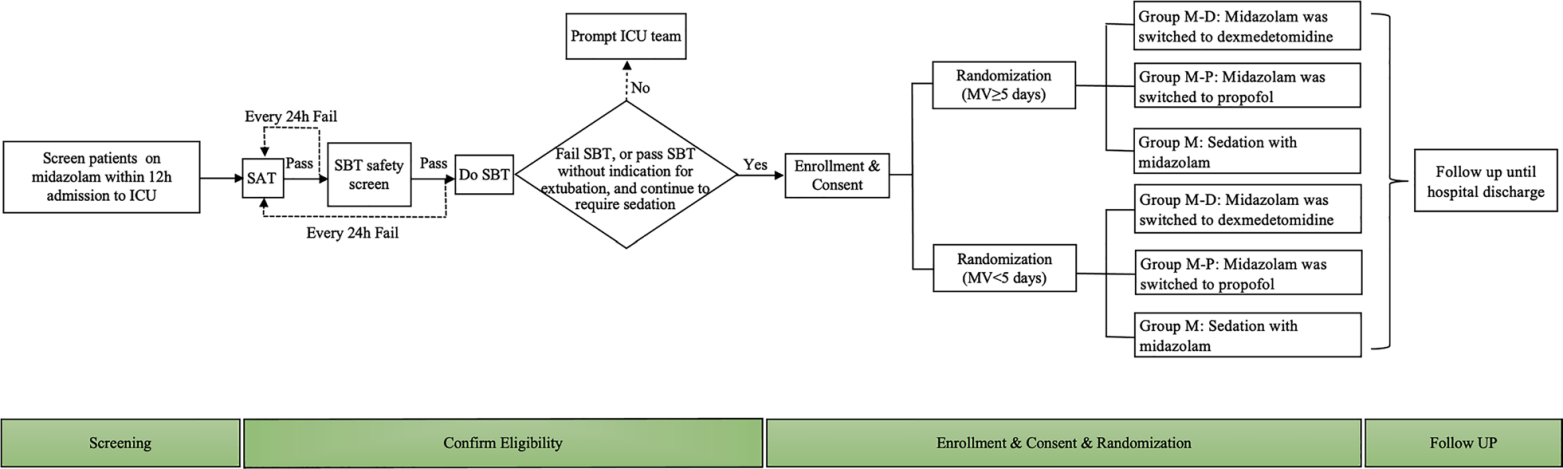
Study protocol. *SAT* spontaneous awakening trial, *SBT* spontaneous breathing trial

If patients failed the SBT trial or passed the SBT without indication for extubation and continued to require sedation assessed by the treating physician, they would be confirmed as eligible and included. Otherwise, they would be excluded (Fig. 1).

### Randomization

Random sequence was computer-generated in permuted blocks of 6 participants, with stratification according to the duration of mechanical ventilation (≥ 5 days or < 5 days), which was concealed in a consecutively numbered, sealed, opaque envelope by one study personnel. Another study personnel opened the envelope before each assignment. Eligible patients were randomly assigned 1:1:1 to group M-D, group M–P, and group M.

### Intervention

In group M, patients continued current midazolam with a maintenance dose of 0.04 to 0.20 mg/kg/h. In group M-P, midazolam was switched to propofol with a continuous maintenance infusion of 0.50–3.00 mg/kg/h. In group M-D, midazolam was switched to dexmedetomidine with a continuous maintenance dose of 0.2–0.7 µg/kg/h. If insufficient sedation, the maximum dosage of up to 1.4 µg/kg/h was permitted. Bedside nurses titrated sedatives to maintain the target sedation level (RASS score − 2 to 0) and assessed sedation depth every 4 h (or more frequently when indicated) using the RASS score [37].

After enrollment, all the patients continued to be managed with an SAT and SBT protocol by the physician and respiratory therapist every morning [37] (details in S4.5, Additional file 1). If patients passed the SBT trial, physicians and respiratory therapists jointly decided to extubate patients. What’s more, whenever the patient’s condition deteriorated, the patient could withdraw from the study at the physician’s discretion.

### Outcomes

The primary outcome was weaning time, defined as the period from randomization to extubation. Secondary outcomes included recovery time from sedation cessation until awakening, extubation time from sedation stopping to extubation, incidence and duration of delirium, length of ICU and hospital stay, percentage of time that RASS scores were within the target sedation range, ICU and hospital mortality, and adverse events [23, 38]. Sedation-related costs (acquisition cost of sedatives and ICU treatment cost) were an exploratory outcome (details described in S4.6, Additional file 1).

### Statistical analysis

Weaning time is the primary outcome. Previous studies showed that the means (± standard deviations) for midazolam, propofol, and dexmedetomidine were 97.9 ± 54.6 h, 34.8 ± 29.4 h and 24.2 ± 1.67 h in long-term sedation, respectively [11, 25]. Considering the huge differences in weaning time among these medications and the fairly minor difference between propofol and dexmedetomidine, we assumed that weaning time was 34.8 h in group M-P and that it would be reduced by 12 h in group M-D with clinical significance, calculating the standard deviation (25.2 h) by combining variances of propofol and dexmedetomidine. A sample size of 213 from three groups was thus estimated to give 80% power and a two-sided significance level of 0.05. Some patients possibly withdrew the treatment, and 252 patients were enrolled to manage a 15% dropout rate.

Data were primarily analyzed following the intention-to-treatment (ITT) principle. Post hoc analyses, including per-protocol (PP) and subgroup analyses, were performed. The treatment protocols (group M–D, group M–P, or group M) were introduced as two dummy variables to obtain hazard ratios, odds ratios, or mean difference for comparison with the reference group.

Assessments of between-group differences of outcomes were described in S4.8, Additional file 1.

## Results

### Participants and baseline characteristics

**Fig. 2:**
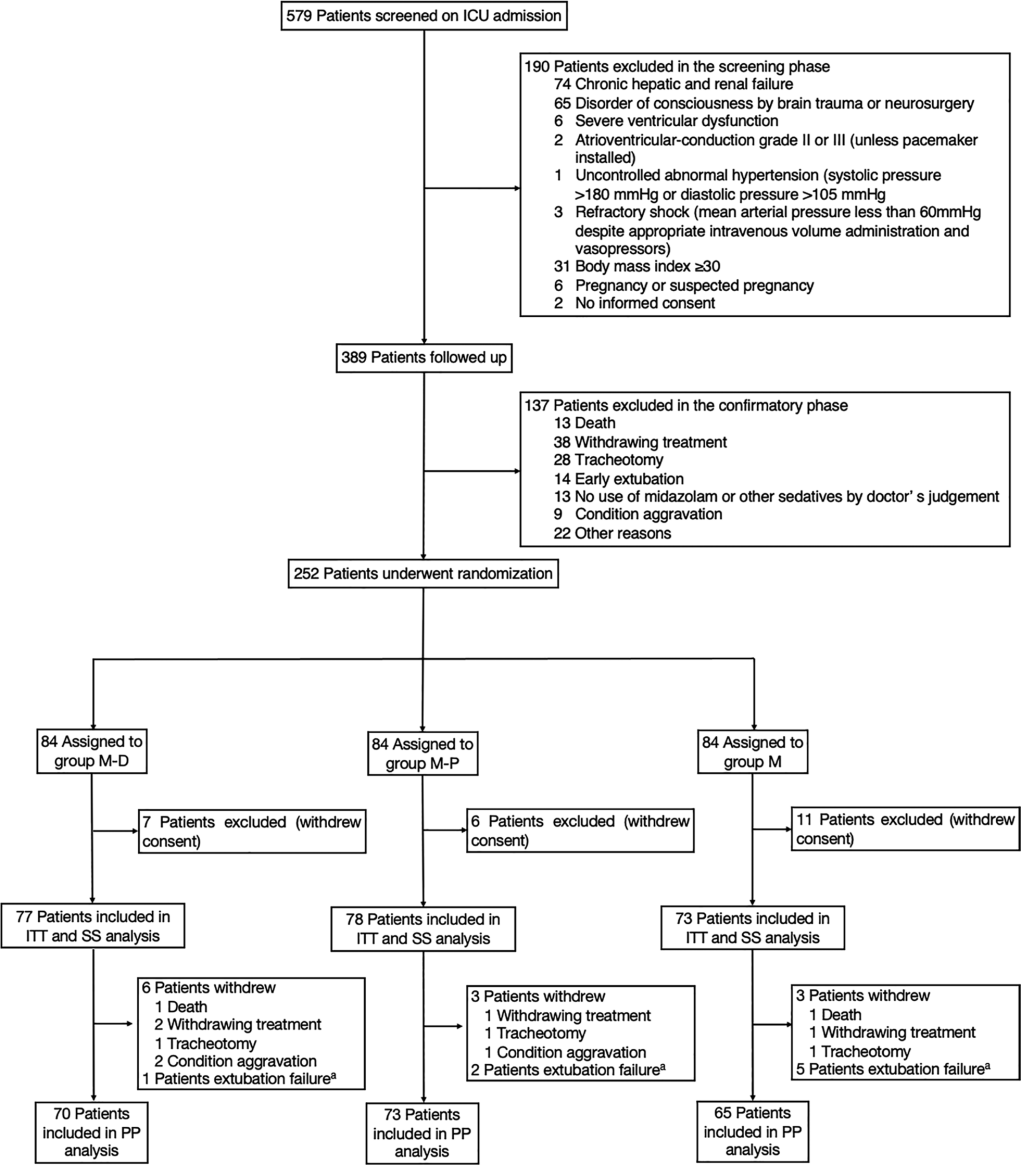
Patients screened, enrollment, randomization, and treatment flow diagram. ^a^Extubation failure: patients received reintubation within 48 h after extubation. *ITT* intention-to-treat analysis, *SS* safety set analysis, *PP* per-protocol analysis

Five hundred seventy-nine patients were initially screened, and 252 patients with confirmed eligibility were randomized with stratification for mechanical ventilation duration (≥ 5 days or < 5 days). Two hundred twenty-eight patients were included in the ITT analysis, and 208 patients completed the study protocol. There were 77 patients in group M–D, 78 patients in group M–P, and 73 patients in group M, respectively (Fig. 2). Baseline characteristics at admission to ICU and enrollment were similar among the groups (Table 1), and the percentage of evaluation times within the individual target sedation range of all the patients was 84.2% before enrollment (eFigure 1 in Additional file 2).

**Table 1:** Baseline characteristics of patients in intention-to-treat analysis

Characteristic	1 Group M–D (n = 77)	2 Group M–P (n = 78)	3 Group M (n = 73)
*Characteristics at admission to ICU*			
Age, mean (SD), year	54.5 (14.5)	51.0 (16.0)	50.8 (15.4)
Male, No. (%)	50 (64.9)	56 (71.8)	52 (71.2)
BMI, mean (SD), kg/m^2^	23.3 (3.3)	24.2 (4.0)	22.9 (3.5)
Apache II score, median (IQR)	20 (15–25)	20 (16–25)	21 (17–25)
SOFA score, median (IQR) ^a^	6 (4–9)	6 (4–9)	6 (4–8)
RASS score, median (IQR)	− 3 (− 4 to 2)	− 3 (− 4 to 3)	− 3 (− 4 to 2)
*Diagnosis at ICU admission, No. (%)*			
Sepsis	19 (24.7)	19 (24.4)	15 (20.5)
Pneumonia	12 (15.6)	17 (21.8)	15 (20.5)
Severe acute pancreatitis	22 (28.6)	18 (23.1)	16 (21.9)
Trauma	12 (15.6)	13 (16.7)	11 (15.1)
Other diseases	12 (15.6)	11 (14.1)	16 (21.9)
*Admission source of patients, No. (%)*			
Emergency department	31 (40.3)	41 (52.6)	25 (34.2)
Medical ward	16 (20.8)	14 (17.9)	14 (19.2)
Surgical ward	10 (13.0)	9 (11.5)	16 (21.9)
Postoperative room	20 (26.0)	14 (17.9)	18 (24.7)
PaO_2_:FiO_2_, median (IQR), mmHg	192.3 (104.2–274.8)	168.1 (97.0–252.0)	188.5 (110.7–255.5)
Use of vasopressors, No. (%)	30 (39.0)	29 (37.2)	29 (39.7)
*Characteristics at enrolled*			
SOFA score, median (IQR) ^a^	4 (3–7)	5 (3–7)	5 (3–8)
RASS score, median (IQR)	− 3 (− 3 to 2)	− 3 (− 3 to 2)	− 3 (− 3 to 2)
Duration of MV, median (IQR), day	4.7 (3.3–6.9)	4.6 (2.7–6.8)	4.6 (2.4–6.7)
*Duration of MV, No. (%)*			
≥ 5 days	43 (55.8)	41 (52.6)	39 (53.4)
< 5 days	34 (44.2)	37 (47.4)	34 (46.6)
Duration of sedation, median (IQR), day	3.7 (1.9–6.8)	3.9 (2.4–6.1)	4.0 (1.9–6.6)
The maintenance dose of midazolam, median (IQR), mg/kg/h	0.060 (0.050–0.083)	0.067 (0.060–0.090)	0.070 (0.042–0.090)
The accumulated dose of midazolam, median (IQR), mg	400.0 (200.0–800.0)	446.5 (200.0–700.0)	400.0 (150.0–900.0)
The maintenance dose of fentanyl, median (IQR), μg/kg/h	0.770 (0.600–0.900)	0.700 (0.600–0.840)	0.800 (0.600–1.020)
The accumulated dose of fentanyl, median (IQR), mg	5.0 (3.0–9.0)	4.5 (3.2–7.0)	4.4 (2.5–10.5)
Triglyceride, median (IQR), mmol/L	1.6 (1.2–2.0)	1.6 (1.2–2.2)	1.5 (1.1–2.0)
Delirium, No.(%)^b^	3 (3.9)	2 (2.6)	2 (2.7)

ICU, Intensive Care Unit; BMI, Body Mass Index; APACHE II, Acute Physiology and Chronic Health Evaluation II; IQR, Interquartile Range; SOFA, Sequential Organ Failure Assessment; RASS, Richmond Agitation and Sedation Scale; FiO_2_, fraction of inspired oxygen; PaO_2_, partial pressure of arterial oxygen; MV, Mechanical Ventilation

^a^SOFA score excluded the central nervous system score (range of values were 0–20, and higher score indicated greater illness)

^b^Calculated from patient ICU admission to enrollment, and delirium assessed if RASS greater than − 3 by use of the Confusion Assessment Method for the ICU (CAM-ICU)

### Study outcomes

**Fig. 3:**
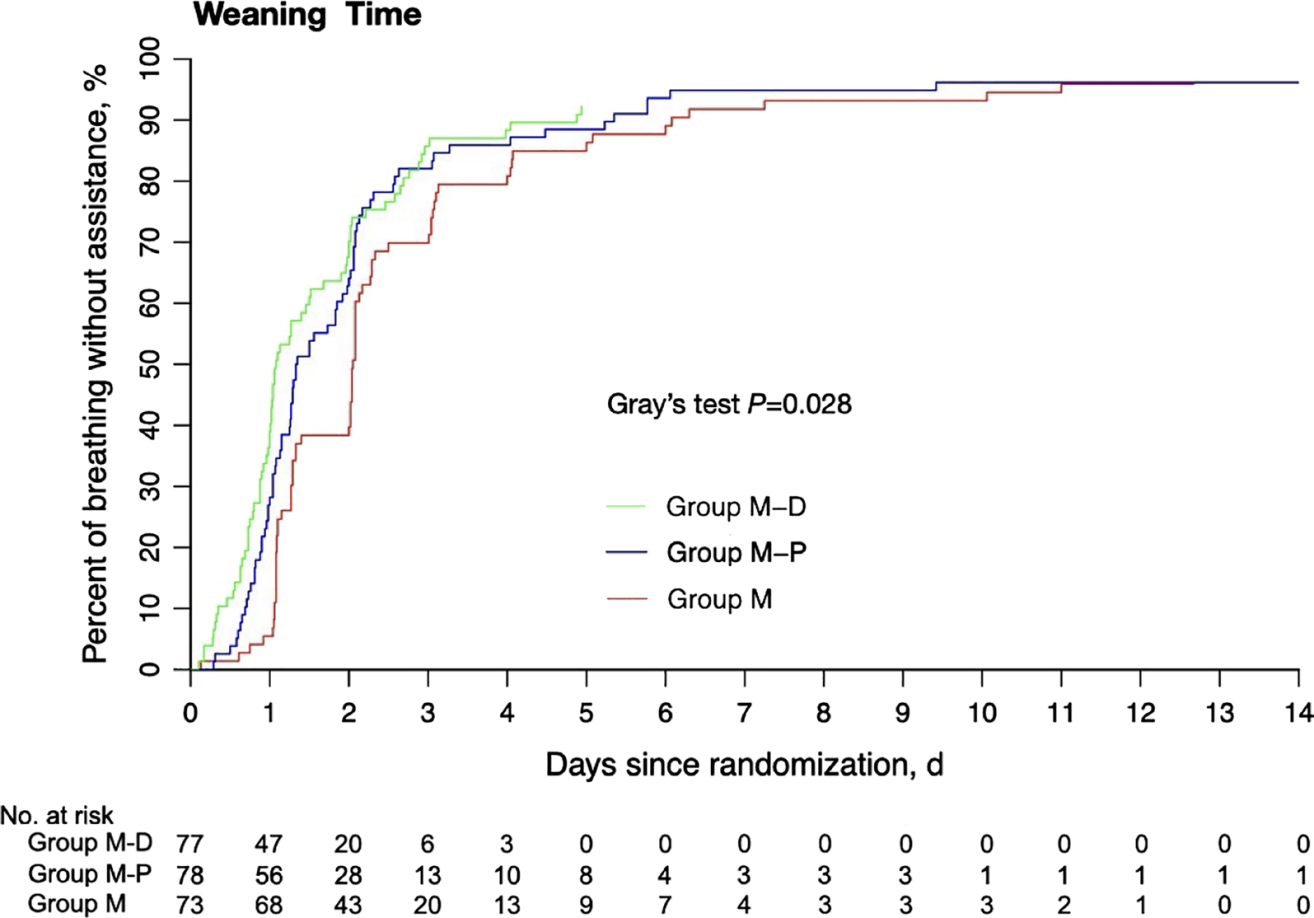
Weaning time in group M–D, group M–P, and group M

Using ITT analysis, recovery time and extubation time in group M–D were shorter than in group M–P (both *P* < 0.001), while no significant difference was observed in the weaning time (*P* = 0.610); compared with group M, patients in group M-D had distinctly less weaning time (25.0 h versus 49.0 h; HR 1.47, 95%CI 1.05 to 2.06; *P* = 0.025) and a lower incidence of delirium (19.5% versus 43.8%; OR 0.31, 95%CI 0.15 to 0.63; *P* = 0.002), and also required a shorter time to recovery and extubation (*P* < 0.001) (Table 2 and Fig. 3); in comparison with group M, patients in group M-P experienced earlier recovery (*P* < 0.001), extubation (*P* < 0.001), and weaning (*P* = 0.048) (Table 2 and Fig. 3). In addition to an earlier discharge from ICU in group M-D than in group M (14.8 days [IQR, 9.9–18.5] versus 17.9 days [IQR, 10.5–23.8], *P* = 0.006), the PP analysis showed similar results to the ITT analysis (Table S1 in Additional file 2). In the subgroup analyses of mechanical ventilation ≥ 5 days or less, patients in group M required a longer time to recovery and extubation than the other two groups (*P* < 0.05); Tables S2 and S3, Additional file 2). No significant difference was observed in successful extubation, tracheotomy, duration of delirium, and death during the ICU and hospital stay among the groups (Table 2, eFigure 2 in Additional file 2). Changes of heart rate and blood pressure after randomization among the groups were also recorded (eFigure 3 in Additional file 2).

**Table 2:** Study outcomes using the intention-to-treatment analysis

					Value of pairwise comparison		
					1 versus 2	1 versus 3	2 versus 3
Outcome Measure	1 Group M–D (n = 77)	2 Group M–P (n = 78)	3 Group M (n = 73)	*P* value	HR, OR, or difference (95% CI), *P* value	HR, OR, or difference (95% CI), *P* value	HR, OR, or difference (95% CI), *P* value
Weaning time^a^, median (IQR), h	25.0 (18.0–48.0)	32.0 (23.0–51.0)	49.0 (26.5–73.0)	0.028	HR = 1.10 (0.77, 1.58), *P* = 0.610	HR = 1.47 (1.05, 2.06), *P* = 0.025	HR = 1.33 (1.00, 1.78), *P* = 0.048
Recovery time^b^, median (IQR), h	0.3 (0.0–1.5)	2.0 (1.0–4.5)	7.0 (2.0–28.0)	< 0.001	D = − 1.8 (− 1.9, − 1.8), *P* < 0.001	D = − 6.5 (− 6.6, − 6.5), *P* < 0.001	D = − 4.7 (− 4.7, − 4.6), *P* < 0.001
Extubation time^b^, median (IQR), h	0.5 (0.0–6.8)	3.5 (1.5–5.5)	7.8 (2.0–30.2)	< 0.001	D = − 2.5 (− 2.5, − 2.5), *P* < 0.001	D = − 7.3 (− 7.3, − 7.2), *P* < 0.001	D = − 4.8 (− 4.8, − 4.7), *P* < 0.001
Sedation administration duration^b^, median (IQR), h	24.0 (13.3–37.5)	26.0 (20.0–47.8)	25.0 (24.0–45.0)	0.020	D = − 4.3 (− 4.3, − 4.2), *P* < 0.001	D = − 2.2 (− 2.2, − 2.1), *P* < 0.001	D = 2.1 (2.1, 2.2), *P* < 0.001
Percentage of times within target sedation range, %	71.4	42.9	39.2	< 0.001	D = 28.5 (24.1, 32.9),* P* < 0.001	D = 32.2 (27.6, 36.8),* P* < 0.001	D = 3.7 (− 0.9, 8.3),* P* = 0.191
Successful extubation, No. (%)	70 (90.9)	73 (93.6)	65 (89.0)	0.610	NA	NA	NA
Tracheotomy, No. (%)	2 (2.6)	1 (1.3)	4 (5.5)	0.265	NA	NA	NA
Delirium, No. (%)	15 (19.5)	23 (29.5)	32 (43.8)	0.005	OR = 0.58 (0.27, 1.21), *P* = 0.150	OR = 0.31 (0.15, 0.63), *P* = 0.002	OR = 0.54 (0.27, 1.05), *P* = 0.069
The accumulated dose of fentanyl after randomization, median (IQR), mg	1.5 (1.0–2.5)	2.5 (1.0–4.0)	2.0 (1.0–4.0)	0.012	D = − 1.0 (− 1.0, − 1.0), *P* < 0.001	D = − 0.5 (− 0.6, − 0.5), *P* < 0.001	D = 0.5 (0.5, 0.5), *P* < 0.001
The average maintenance dose of sedatives ^c^, median (IQR), mg/kg/h or μg/kg/h	0.247 (0.171–0.392)	0.767 (0.567–0.962)	0.074 (0.053–0.128)	–	–	–	–
ICU duration^a^, median (IQR), day	13.9 (9.9–18.4)	14.2 (9.8–18.9)	18.3 (11.7–28.7)	0.148	NA	NA	NA
Length of hospital stay^a^, median (IQR), day	18.4 (12.5–25.5)	19.4 (14.9–34.5)	23.8 (17.3–40.3)	0.448	NA	NA	NA
ICU mortality, No. (%)	2 (2.6)	7 (9.0)	5 (6.8)	0.241	NA	NA	NA
Hospital mortality, No. (%)	2 (2.6)	7 (9.0)	5 (6.8)	0.241	NA	NA	NA
Adverse effects, No. (%)	5 (6.5)	13 (16.7)	7 (9.6)	0.116	NA	NA	NA
Hypotension, No. (%)	4 (5.2)	2 (2.6)	0 (0.0)	0.148	NA	NA	NA
Bradycardia, No. (%)	1 (1.3)	1 (1.3)	0 (0.0)	1.000	NA	NA	NA
Hypertension^d^, No. (%)	0 (0.0)	9 (11.5)	5 (6.8)	0.004	0.003	0.025	0.321
Tachycardia, No. (%)	1 (1.3)	2 (2.6)	2 (2.7)	0.869	NA	NA	NA
Triglyceride, median (IQR), mmol/L	1.76 (1.15–2.24)	1.85 (1.28–2.37)	1.58 (1.11–2.46)	0.757	NA	NA	NA

OR = odds ratio; HR = hazard ratio; D = difference; IQR, Interquartile Range; ICU, Intensive Care Unit; NA, not applicable

^a^Calculated using competing risk model analysis

^b^A total of 12 patients were excluded in this analysis, including 3 patients in group M (1 death, 1 withdrew treatment, and 1 tracheotomy), 3 patients in group M-P (1 withdrew treatment, 1 tracheotomy, and 1 disease deteriorated), and 6 patients in group M-D (1 death, 2 withdrew treatment, 1 tracheotomy, and 2 disease deteriorated). Recovery time: Time from stopping sedation to awakening. Extubation time: Time from stopping sedation to extubation

^c^The average dose of midazolam, propofol, or dexmedetomidine after randomization; mg/kg/h for midazolam or propofol; μg/kg/h for dexmedetomidine

Weaning time: Time from randomization to extubation

^d^Analyzed using chi-squared test or fisher's exact test, with adjustment of significance level to 0.017 for comparisons for any two groups

Sedation administration duration: Sedation administration period after randomization. ICU duration: Time from patient admission to ICU until discharge from ICU. Length of hospital stay: Time from patient screened until discharge from hospital

### Sedation efficacy and administration

After randomization, the percentage of time within the target sedation range in group M–D was higher than those in group M–P (71.4% versus 42.9%, *P* < 0.001) and group M (71.4% versus 39.2%, *P* < 0.001), while there was no significant difference between group M–P and group M (Table 2 and eFigure 4). Patients in group M–D required less fentanyl and a shorter duration of sedative administration than the other two groups (all *P* < 0.001); variables as mentioned above in group M–P were more than those in group M (*P* < 0.001; Table 2). Only three patients in group M–D received dexmedetomidine exceeding 0.7 µg/kg/h, and no one received additional sedatives. Sedative acquisition cost in group M–P was more expensive than in group M and group M-D (both *P* < 0.001), without significant difference in ICU treatment cost among the three groups (Table S4 in Additional file 2).

### Adverse events

There was no significant difference in hypotension and bradycardia among the groups. Hypotension occurred in four patients (5.2%) and bradycardia in one patient (1.3%) in group M–D; hypotension in two patients (2.6%) and bradycardia in one patient (1.3%) in group M–P; and no one experienced hypotension and bradycardia in group M. A greater incidence of hypertension was observed in group M–P than in group M–D (11.5% vs. 0, *P* = 0.003), without significant difference in tachycardia and hypertriglyceridemia among these groups (Table 2, and Tables S5, S6 in Additional file 2).

## Discussion

To our knowledge, this is the first study to investigate the sequential use of midazolam and dexmedetomidine combined with the ventilator weaning process in critically ill, mechanically ventilated patients. Applying light sedation with nonbenzodiazepine drugs to practice and achieving optimum sedation in long-term sedation remain a clinical challenge so far since each sedative agent possesses specific risks and benefits. Dexmedetomidine is becoming increasingly popular due to its “cooperative sedation” and opioid-sparing properties, and the absence of respiratory depression. Nevertheless, the SPICE III trial [28] reported that early use of dexmedetomidine in mechanically ventilated patients did not improve outcomes more than usual care. Most patients required deep sedation and received supplemental sedatives: propofol, benzodiazepines, or both. Up till now, midazolam is quite commonly used for providing effective sedation and amnesia in selected mechanically ventilated patients and certain low-resource areas [5–7, 28, 31, 32]. However, the issues regarding its accumulation effect and increased risk of delirium occurrence were always concerning. In our previous study, sequential use of midazolam and propofol improved outcomes and showed better cost–benefit ratio than midazolam or propofol used alone [33]. Therefore, based on choosing the right timing and appropriate sedatives according to the pharmacological properties of sedatives throughout the disease progress, our institution established a strategy-sequential use of midazolam and dexmedetomidine combining ventilator weaning process for the critically ill, mechanically ventilated patients receiving midazolam determined by the treating physician, aiming to maximize the advantages of and avoid adverse effects of midazolam and dexmedetomidine.

The main findings of the present study were as follows: (1) Sequential use of midazolam and dexmedetomidine was associated with a shorter time to recovery and extubation, more time in the target sedation range, lower fentanyl requirements, and a lower acquisition cost of sedation than sequential use of midazolam and propofol, while the weaning time was similar. (2) It was associated with faster recovery, earlier extubation, shorter weaning time, more time in the target sedation range, reduced fentanyl requirements, and lower delirium rate compared with midazolam alone. (3) In addition, in comparison with midazolam alone, sequential use of midazolam and propofol provided a similar sedation quality and facilitated earlier recovery, extubation, and weaning, but it increased the cost of sedative after randomization.

In accordance with previous studies [23, 24], the goal of sedation target level was a RASS score of 0 to − 2 after randomization, and the proportion of time at target sedation level (72.6%) in group M-D was remarkably higher than in group M–P (45%) and group M (39.7%). This finding suggested that dexmedetomidine used during the weaning period was easier to achieve light sedation than propofol and midazolam. Patients sequentially treated with midazolam and dexmedetomidine also required a lower cumulative dose of fentanyl, probably resulting from its analgesic effect. The reduced use of fentanyl possibly contributed to lowering the risk for occurrence of delirium. Previous studies reported that among critically ill patients who received early dexmedetomidine for sedation, most of them (61% or 85%) failed to achieve the prescribed level of sedation and received supplemental sedatives despite remaining at the maximum dosage of 1.4 µg/kg per hour or 2.5 µg/kg per hour, and hypotension and bradycardia were significantly more common in the dexmedetomidine group [19, 20, 23, 24, 26, 28, 31]. However, in the present study, not only did patients receive a much lower dosage of dexmedetomidine, within the range of 0.2 to 0.7 µg/kg/h except for three patients receiving dexmedetomidine exceeding 0.7 µg/kg per hour, and no one received additional sedatives, but also hypotension and bradycardia were rarely observed during the study period. Possibly because midazolam was replaced by dexmedetomidine was at the relatively stable stage of disease—the weaning period—dexmedetomidine was administrated with a relatively low maintenance dose, and thus it was not commonly associated with cardiovascular side effects. This finding implied that midazolam could be smoothly switched to dexmedetomidine, thereby achieving light sedation easily and safely during the weaning period.

In the present study, midazolam and dexmedetomidine used sequentially were associated with earlier recovery and extubation than the sequential use of midazolam and propofol. In the PRODEX trial, dexmedetomidine still led to earlier extubation than propofol [24]. Dexmedetomidine associated with more time at light levels of sedation might expedite recovery and extubation, while this effect had a slight influence on liberation from ventilator, and thus group M-D and group M-P both experienced similar weaning time. Compared with midazolam alone, sequential use of midazolam and dexmedetomidine facilitated earlier recovery, extubation, and liberation from ventilator, and reduced the incidence of delirium. These findings were consistent with the results of the SEDCOM and MIDEX trials [23, 24]. Possible explanations were that dexmedetomidine-driven light sedation as early as possible during the weaning period resulted in less use of and a lower accumulative effect of midazolam, and thus led to earlier awakening, spontaneous breathing, and extubation, as well as facilitating weaning, and improving the delirium outcome [2, 4, 16, 39]. Less delirium with dexmedetomidine may have contributed to a shorter time to extubation and earlier discharge from ICU [40, 41]. However, the SPICE III study [28] showed that early use of dexmedetomidine in critically ill patients was not superior to the usual care (midazolam, propofol, or other sedatives) in primary outcomes (90-day mortality, ventilator-free days, and coma-free days). This finding indicated that the impact of the timing of using dexmedetomidine on outcomes of critically ill, mechanically ventilated patients might need to be further explored.

As the conception of light sedation has been widely accepted in recent years, sedatives have been titrated to achieve the goal of light sedation in our usual care whenever possible. In this study, patients sequentially treated with midazolam and propofol still showed faster recovery, extubation, and weaning than midazolam alone, similar to the results of our previous study [33]. This reinforced the importance of the use of an appropriate sedation strategy for patients undergoing prolonged mechanical ventilation. In addition, the acquisition cost of sedative was more expensive than midazolam used alone and midazolam and dexmedetomidine used sequentially. However, this finding was not suitable for generalizability since the price of sedatives varied widely in different countries.

This study has several limitations. First, this trial was unblinded, as the appearance of propofol was different from midazolam and dexmedetomidine. However, almost 300 nurses were randomly involved in the care of all the patients, and respiratory therapists managed them with the routine ventilator weaning protocol. Second, this was a single-center, randomized, controlled trial, limiting the generalizability, and further research is needed to validate this strategy’s efficacy. Lastly, the use of midazolam before enrollment was not in accordance with the PAD 2013 and PADIS 2018 clinical practice guidelines [2, 30]; however, midazolam use in ICUs was quite common, especially in selected mechanically ventilated patients [5–7, 28, 31, 32]. Additionally, different sedative drugs and sedation durations before randomization might influence outcomes. Therefore, we only screened mechanically ventilated patients initially treated with midazolam as determined by the managing physician and randomized them with stratification by mechanical ventilation duration (≥ 5 days or less), as mechanical ventilation duration was close to the sedation administration time, to ensure the homogeneity of the population among the groups.

## Conclusions

In critically ill, mechanically ventilated patients initially receiving midazolam at the physician’s discretion, the sequential use of midazolam and dexmedetomidine required a shorter time to recovery and extubation, and a lower dosage of fentanyl than the patients treated with midazolam and propofol sequentially; this was also associated with faster recovery, earlier extubation, shorter weaning time, more time at the target sedation level, lower fentanyl requirements, and less delirium than midazolam alone, without increasing adverse events. This finding indicated that the sequential use of midazolam and dexmedetomidine for long-term sedation was an effective and safe sedation strategy and might provide clinically relevant benefits for selected critically ill, mechanically ventilated patients.

## Supplementary Information

**Additional file 1.** Trial protocol and statistical analysis plan.

**Additional file 2.** Statistical analysis method and data supplement related to sedation, delirium, study outcomes by the per-protocol analysis and subgroups analysis, sedation-related costs, heart rate, and blood pressure.

## Data Availability

The datasets used for the analysis in the current study are available from the corresponding author on reasonable request. Yan Kang and Yongfang Zhou had full access to all the data in the study and take responsibility for the integrity of the data and the accuracy of the data analysis.

## Abbreviations

SAT: Spontaneous Awakening Trial
SBT: Spontaneous Breathing Trial
RASS: Richmond Agitation-Sedation Scale
ITT: Intention-To-Treatment
PP: Per-Protocol
