# Supplementary material for: Sequential use of midazolam and dexmedetomidine for long-term sedation may reduce weaning time in selected critically ill, mechanically ventilated patients: a randomized controlled study
Source: Crit Care. 2022 May 3;26:122. doi: 10.1186/s13054-022-03967-5 (PMC9066885; doi:10.1186/s13054-022-03967-5)
Supplement: Supplementary file 1 — Additional file 1. Trial protocol and statistical analysis plan. [file 13054_2022_3967_MOESM1_ESM.docx]

**Sequential Use of Midazolam and Dexmedetomidine for Long-term Sedation May Reduce Weaning Time in Selected Critically Ill, Mechanically Ventilated Patients: A Randomized Controlled Study**

**Yongfang Zhou ^1^, M.M. Jie Yang ^1^, M.M. Bo Wang ^1^, PhD. Peng Wang ^1^, B.S. Zhen Wang ^1^, B.S. Yunqing Yang ^1^, B.S. Guopeng Liang ^1^, M.M. Xiaorong jing ^1^, B.S. Xiaodong Jin ^1^, MD. Zhongwei Zhang ^1^, MD. Yiyun Deng ^1^, PhD. Chenggong Hu ^1^, PhD. Xuelian Liao ^1^, PhD. Wanhong Yin ^1^, PhD. Zhihong Tang ^1^. MS. Yongming Tian ^1^, M.M. Liyuan Tao ^2^, PhD. Yan Kang ^1*^, MD.**

**Author affiliations:  ^1^ Department of Critical Care Medicine, West China Hospital of Sichuan University, Chengdu, Sichuan 610041, China. ^2^Research Center of Clinical Epidemiology, Peking University Third Hospital, Beijing 100191, China.**

**^*^ Correspondence author: Yan Kang, MD**

**Address: Guoxue Alley 37#, Wuhou District,**

**Chengdu, Sichuan Province, 610041.**

**Email:** [**kangyan@scu.edu.cn**](mailto:kangyan@scu.edu.cn)**.**

**Additional file Treatment protocol**

**Contents**

**Additional file S1. INTRODUCTION.................................................Section.1**

**Additional file S2. HYPOTHESIS......................................................Section.2**

**Additional file S3. OBJECTIVES......................................................Section.3**

**Additional file S4. METHODS..........................................................Section.4**

**Study design.......................................................................** **S4.1**

**Patients................................................................................S4.2**

**Screening phase ...................................S4.2.1**

**Eligibility confirmation........................S4.2.2**

**Randomization method and allocation concealment ........S4.3**

**Intervention...........................................................................S4.4**

**Weaning and extubation......................................................S4.5**

**Endpoints..............................................................................S4.6**

**Sample size calculation........................................................S4.7**

**Statistical analysis plan........................................................S4.8**

**Treatment protocol**

**S1. INTRODUCTION**

Sedation in critically ill mechanically ventilated patients is a key component of bedside care in intensive care units (ICU), maximizing patient comfort and tolerability of interventions, preventing unplanned removal of lines and catheters and reducing metabolic demands ^1, 2^. Current sedatives have different side-effect profiles and remain problematic in long-term sedation as result of different mechanisms of action ^3^. Midazolam and propofol have been the most commonly sedative drugs used for ICU patients ^4-6^. Midazolam accumulates unpredictably, may delay recovery and extubation, and is a risk factor for delirium ^3, 7-10^. Propofol, a sedative-hypnotic agent, is associated with dose-dependent effects and fast recovery without accumulation ^3，7，8，10^. However, high-dose or prolonged propofol use may cause hypertriglyceridemia, fatal propofol infusion syndrome, and cardiorespiratory depression ^3, 8, 10-13^.

In contrast to traditional sedative medications, dexmedetomidine, a highly selective central alpha-2 adrenergic agonist is an anxiolytic, sedative, and analgesic medication, and notable for its ability to provide cooperative or semi-arousable sedation without risk of respiratory depression ^3，14, 15^. Several studies reported that dexmedetomidine is suitable for light to moderate sedation, and when used for prolonged sedation (＞24 hours) is as effective as midazolam or propofol for prolonged sedation (＞24 hours) ^16，17^. Dexmedetomidine reduced the duration of mechanical ventilation, incidence of delirium and opioids dosage, associated with more pharmaceutic cost but reducing the total ICU treatment cost compared with midazolam, and improving patients’ ability to communicate pain compared with midazolam and propofol ^18-24^. However, previous studies reported dexmedetomidine was more applicable for light to moderate sedation than deep sedation despite the use of the maximum dose of dexmedetomidine (1.4ug/kg/hour) ^16-18^, and was associated with markedly increased fentanyl needs in patients with RASS target -3 or deeper ^23^; what’s more, bradycardia and hypotension were more common with dexmedetomidine ^18-19, 21^. In view of the literatures and real clinical practice, midazolam is still frequently used, particularly when deep sedation (RASS score of −3 or less) was indicated and in limited-resource countries, or in some selected patients. ^19, 24^

In view of the advantages and disadvantages of different sedative drugs, clinician could take account of the pharmacological properties of sedative drugs as well as the individual patients’ characteristics and needs, and the requirements for respiratory support and sedation could probably be synchronized based on the disease progress. Our previous study showed that sequential use of midazolam and propofol according to ventilator weaning process was associated with faster recovery and extubation compared with midazolam, and lower [pharmaceutic](file:///C:\Program%20Files%20(x86)\Youdao\Dict\7.1.0.0421\resultui\dict\?keyword=pharmaceutic) cost compared with propofol ^25^. So far there is still lack of research regarding sequential use of midazolam and dexmedetomidine based on ventilator weaning process. Sequential use of midazolam and dexmedetomidine for prolonged sedation combining ventilator weaning process may have distinct advantages.

**S2. HYPOTHESIS**

It is hypothesized that a paired protocol of sequential sedation and ventilator weaning process (sequential use of midazolam and dexmedetomidine based on ventilator weaning process) can improve outcomes in mechanically ventilated patients.

**S3. OBJECTIVES**

To test our hypothesis, we plan to assess the efficacy, safety and cost of sequential use of midazolam and either dexmedetomidine or propofol, or midazolam used alone for long-term sedation in critically ill, mechanically ventilated patients.

**S4. METHODS**

**S4.1 Study design**

A single center, randomized, controlled trial with allocation concealment and intention to treatment analysis, comparing sequential use of midazolam and dexmedetomidine, or sequential use of midazolam and propofol, or midazolam used alone for long-term sedation in critically ill, mechanically ventilated patients.

**S4.2 Patients**

**Patients intubated, undergoing mechanical ventilation will be enrolled through two phases - a screening phase and a** **confirmatory phase (Figure 1).Figure. 1**

**
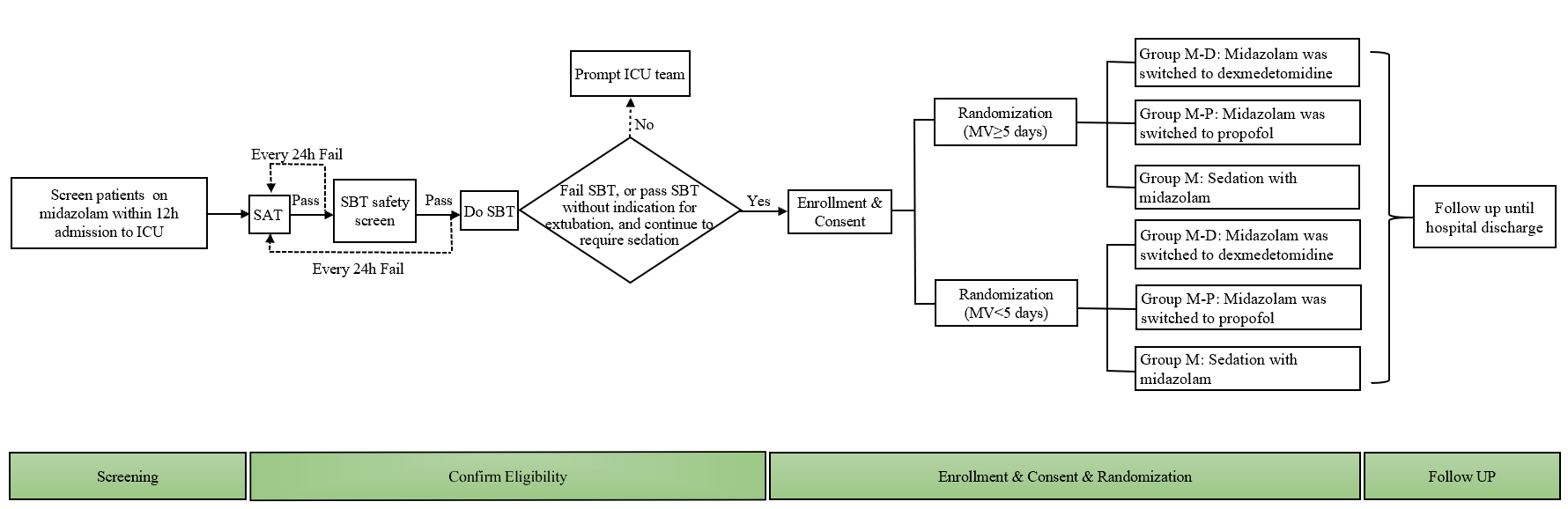
**

**S4.2.1 Screening phase**

During the screening phase, all intubated, mechanically ventilated patients will be screened for initial inclusion and exclusion criteria within 12 hours of admission to ICU.

The initial inclusion criteria were age between 18 years old and 80 years old, with an anticipated mechanical ventilation of ≥72 hours; fentanyl is used for analgesia and midazolam for sedation, with titration to achieve the individual sedation target level, determined by treating physician.

The exclusion criteria including known or suspected allergy to midazolam, propofol or dexmedetomidine; pregnancy or suspected pregnancy; history of alcoholism or intake of anti-anxiety drugs or hypnotics; body mass index≥30; chronic hepatic failure (chronic liver disease decompensation-premorbid Child-Pugh B or C); chronic renal failure (a persistent impairment of kidney function, abnormally elevated serum creatinine for more than 3 months or calculated glomerular filtration rate (GFR) less than 60 ml per minute/1.73m^2^); disorder of consciousness by brain trauma or neurosurgery or unknown etiology or status epilepticus; dialysis of all types; heart rate less than 50/min, atrioventricular-conduction grade II or III (unless pacemaker installed); refractory shock (mean arterial pressure less than 60mmHg despite appropriate intravenous volume administration and vasopressors); moribund state; patients in palliative care only.

Once patient pass the screening phase, study personnel will follow them up every day.

Patients who pass the screening phase, will continue to receive fentanyl for analgesia with maintenance infusion of 1 to 2 μg/kg/hour with titration to achieve the target analgesia level (CPOT score 0-2)^26^, and continue to be treated with midazolam with maintenance infusion of 0.04 to 0.20 mg/kg/hour, titrated to achieve the individual sedation target level. The individual sedation target will be daily determined at the physician’s discretion. Although most of mechanically ventilated patients receiving light to moderate sedation with a target sedation range (RASS score 0 to -2) may be appropriate, but some severely ill patients experiencing ventilator dyssynchrony or requiring aggressive ventilator settings (e.g. ARDS) may require deep sedation (RASS -3 or lower).

All the patients will be managed with a daily spontaneous awakening trial (SAT) followed by a spontaneous breathing trial (SBT) throughout the mechanical ventilation period ^27^.

**S4.2.2 Confirmation phase**

During the confirmatory phase, from the next day morning after the initial screening, clinician will manage patients with the spontaneous awaking trial (SAT), and once patients pass the SAT, respiratory therapists will assess patients using SBT safety screen. Study personnel also will follow them up every day.

In accordance with the SAT protocol, all patients with RASS score＜-2 are assessed with an SAT safety screen by the managing clinician every morning. Patients who pass the screen when there are no contraindications, including severe hypoxemia, myocardial ischemia, hypertensive crisis, status asthmaticus, or sustained agitation with increased use of sedative drugs, will undergo an SAT. ^27^ The analgesic and sedative infusion will be interrupted until patients awakening. Analgesics for active pain will be continued. Patients pass the SAT if they can do the following three simple tasks: opening eyes, squeezing the hand and moving fingers, and expressing discomfort. Patients fail the SAT if they develop sustained agitation, marked dyspnea, SPO_2_<88% for ≥5 minutes or arrhythmias. When patients fail an SAT, bedside nurses will restart analgesics and sedatives at a half of the previous dose and then titrate the medications to achieve the target sedation range. These patients will be reassessed the following morning. While patients with RASS score -2 to 0 will be directly stopped infusion of sedative and analgesics without a SAT, and analgesics for active pain will also be continued. Patients who pass the SAT or those with RASS score -2 to 0 who are interrupted sedation will be immediately managed with the SBT protocol by respiratory therapist.

Patients pass SBT safety screen when they show the underlying causes for respiratory failure resolved or partly resolved, adequate oxygenation (oxygen partial pressure ≥60 mmHg, fraction of inspired oxygen≤50%, and positive end-expiratory pressure ≤10 cmH2O), stable cardiovascular status with no evidence of myocardial ischemia and hypotension, and no or minimal use of vasopressors (dopamine or dobutamine ≤5μg/kg/minute or norepinephrine ≤2μg/minute) ^27^. Subsequently, they will undergo a 30-min SBT trial with pressure support 5~8 cmH_2_O, positive end-expiratory pressure 5 cmH_2_O and fraction of inspired oxygen 40% ^28^. Patients fail a SBT trial when they show any of the following failure signs: Respiratory rate >35 breaths/minute or <8 breaths/ minute, hypoxemia (SPO_2_ or SaO_2_ <90%), abrupt changes in mental status, unstable cardiovascular status with heart rate and blood pressure changing more than 20% from the previous level, or acute cardiac arrhythmia, tachycardia (>140 beats/minute) or bradycardia (<60 beats/ minute), shortness of breath, signs of increased work of breath such as use of accessory muscles or abdominal paradox.

Patients pass the SBT if they don’t exhibit any of aforementioned failure signs followed by extubation, or patients fail the SBT with no sedation indicated, they will be excluded and managed with our local routine protocol. If patients who fail the SBT trial, or pass the SBT without indication for extubation, are re-ventilated at previous support level, exhibiting agitated, anxiety, and discomfort, and continue to require sedation assessed by treating physician, they will be confirmed eligibility. (Figure 1)

**S4.3 Randomization method and allocation concealment**

As the weaning time might be influenced by the duration of sedation administration and mechanical ventilation before randomization, and mechanical ventilation time is close to the duration of sedation, we choose 5 days of mechanical ventilation from screening to enrollment as boundary layer according to the average duration (5 days) of mechanical ventilation in the previous studies.

Random sequence will be electronically generated by a computer in permuted blocks of 3 participants, with stratification by the duration of mechanical ventilation before randomization (≥ 5 days or＜5 days). Random sequence will be concealed in a consecutively numbered, sealed, opaque envelope by one study personnel and the envelope will be opened by another one before each assignment. Eligible patients will be randomly assigned 1:1:1 to receive continuous current sedative drug, sequential use of midazolam and dexmedetomidine (group M-D), sequential use of midazolam and propofol (group M-P), or midazolam used alone (group M).

**S4.4 Intervention**

After randomization, the informed consent is obtained from legally authorized representatives. Eligible patients will be treated with sequential use of midazolam and dexmedetomidine (group M-D), sequential use of midazolam and propofol (group M-P), or midazolam alone (group M).

In group M-D, midazolam is switched to dexmedetomidine with the approved, continuous maintenance dose of 0.2-0.7ug/kg/h, the maximum dose up to 1.4 µg/kg per hour will be permitted when insufficient to achieve the target sedation level, according to the current evidence. Study drugs are titrated to maintain the desired sedation level (RASS score -2 to 0) by bedside nurses. Patients who can’t be adequately sedated with the maximum dose of study drugs, can receive additional sedative drugs for rescue sedation at clinician’s discretion. Sedation depth is assessed every 4 hours (or more frequently when indicated) using RASS score^29^.

In group M-P, midazolam is switched to propofol with a continuous maintenance infusion of 0.50-3.00mg/kg/h.

In group M, patients continue current sedative, midazolam.

The RASS score^29^ ranges from -5 to +4 listed as follows: -5- unarousable by physical stimulation; -4 deep sedation: no response to voice but movement or eye opening to physical stimulation; -3 moderate sedation: movement or eye opening to voice (no eye contact); -2 light sedation: briefly awakens with eye contact to voice (less than 10 seconds); -1 drowsy: not fully alert, sustained awakening to voice (eye opening/eye contact for at least 10 seconds); 0: alert and calm; +1: restless, anxious but movements not aggressive/vigorous; +2 agitated: frequent non-purposeful movement, fights ventilator; +3: very agitated, pulls or removes tubes or catheters, aggressive; +4 : combative: overtly combative, violent, immediate danger to the staff.

**S4.5 Weaning and extubation**

Starting from the next day after enrollment, patients with RASS score＜-2 in each group are managed with a SAT by physician every morning. Patients pass the SAT safety screen ^27^, when there are no contraindications, including severe hypoxemia, myocardial ischemia, hypertensive crisis, status asthmaticus, or sustained agitation with increased use of sedation drugs. Next, the analgesic and sedative infusion will be interrupted until patients are awake, determined from the following three simple tasks: opening eyes, squeezing the hand and moving fingers, and expressing discomfort. When patients develop sustained agitation, marked dyspnea, SPO_2_<88% for ≥5 minutes or arrhythmias, analgesic and sedative will be restarted at a half of the previous dose and then titrated to achieve the target sedation range. The patients will be reassessed the next morning. However, patients with RASS score-2 to 0, will be directly stopped infusion of sedative and analgesics, without a SAT.

Patients who pass a SAT or those with RASS score -2 to 0 who are interrupted sedation will be immediately managed with SBT safety screen and a 30-minute SBT trial by respiratory therapists. Patients pass SBT safety screen when they show the underlying causes for respiratory failure resolved or partly resolved, adequate oxygenation (oxygen partial pressure ≥60 mmHg, fraction of inspired oxygen≤50%, and positive end-expiratory pressure ≤10 cmH_2_O), stable cardiovascular status with no evidence of myocardial ischemia and hypotension, and no or minimal use of vasopressors (dopamine or dobutamine ≤5μg/kg/minute or norepinephrine ≤2μg/minute) ^27^. Subsequently, they will undergo a 30-min SBT trial with pressure support 5~8 cmH_2_O, positive end-expiratory pressure 5 cmH_2_O and fraction of inspired oxygen 40% ^28^. Patients fail a SBT trial when they show any of the following failure signs: Respiratory rate >35 breaths/minute or <8 breaths/ minute, hypoxemia (SPO_2_ or SaO_2_ <90%), abrupt changes in mental status, unstable cardiovascular status with heart rate and blood pressure changing more than 20% from the previous level, or acute cardiac arrhythmia, tachycardia (>140 beats/minute) or bradycardia (<60 beats/ minute), shortness of breath, signs of increased work of breath such as use of accessory muscles or abdominal paradox.

Patients pass the SBT if they don’t exhibit any of aforementioned failure signs, physicians and respiratory therapists will jointly decide to extubate patients. If patients fail the SBT, they will be immediately restarted to ventilate at the previous ventilator support level and reassessed next morning. What’s more, whenever patient’s condition deteriorate, patient can withdraw from the study by physician’s discretion.

**S4.6 Endpoints**

The primary end point is weaning time. Secondary endpoints include recovery time, extubation time, prevalence and duration of delirium, length of ICU and hospital stay, ICU and hospital mortality, percentage of time that RASS scores within the target sedation range, sedation-related costs (acquisition costs of sedation and total ICU treatment costs), and adverse events. Adverse events (including hypertension, hypotension, bradycardia and tachycardia) will be monitored and recorded from starting of study drug until 48 hours after study drug discontinuation ^18^.

Weaning time is defined as the duration between randomization and extubation.

Recovery time is defined as the duration from sedation cessation until awakening and extubation time from sedation cessation to extubation, respectively.

Length of stay in ICU is defined as from admission to ICU to discharge from ICU.

Length of hospital stay: Time from patient screened until discharge from hospital.

Percentage of time within the target sedation range is calculated as: times that RASS score in the target range/total evaluation times x 100%.

Delirium is assessed every eight hours per day using the Confusion Assessment Method for the ICU (CAM-ICU) from randomization to discharge of ICU ^30^.

Adverse events will be monitored and recorded from randomization until 48 hours after sedatives discontinuation. Severe hemodynamic adverse events will be considered if patients exhibit hypertension with systolic pressure greater than 180mmHg or diastolic pressure greater than 100 mmHg or hypotension with systolic pressure less than 80mmHg or diastolic pressure less than 50mmHg, bradycardia with heart rate less than 50/min, or tachycardia with heart rate greater than 140/min, or a greater than 30% change from baseline heart rate or blood pressure. Serum triglyceride concentrations will be monitored at enrollment and sedation cessation.

**S4.7 Sample size calculation**

The weaning time is the primary outcome in this trial. The previous study showed that means (±standard deviations) of weaning time for midazolam, propofol and dexmedetomidine were 97.9 ± 54.6h, 34.8 ± 29.4h and 24.2 ± 1.67h in long-term sedation, respectively ^10, 20^. Considering there are huge differences in weaning time among these medications and fairly minor difference between propofol and dexmedetomidine, we assume weaning time to be 34.8 hours in group M-P and it would be reduced 12 hours in group M-D with clinical significance, and the standard deviation (25.2 h) is calculated by combining variances of propofol and dexmedetomidine. A sample size of 213 of three groups was thus estimated to give 80% power and a two-sided significance level of 0.05. As some patients possibly withdrew the treatment, 252 patients were enrolled for the study in order to manage a 15% dropout rate.

**S4.8 Statistical analysis plan**

Data will be analyzed following the intention-to-treatment principle. Values of normal distribution will be expressed by mean ± standard deviation, values of non-normal distribution will be expressed as median and interquartile range (IQR), and categorical variables as counts and percentages. For continuous variables analysis, the differences in normally distributed data of the three groups will be compared with one-way analysis of variance and differences between any two groups will be analyzed by Student–Newman–Keuls (SNK) methods, P<0.05 is considered statistically significant. For all other outcomes, the significance level is 0.05 for comparisons of three groups, with adjustment to 0.017 for comparisons for any two groups. Competing risk model will be used to assess the effect of the treatment protocols on weaning time, ICU duration and length of hospital stay. Differences in non-normally distributed variables of three groups will be assessed using Kruskal–Wallis analysis of variance and differences between any two groups will be compared with Mann–Whitney 𝑈 test. Categorical variables will be analyzed using Chi-squared test or Fisher's exact test and significant differences between any two groups will be further analyzed with the P value adjusted to 0.017. Considering in-hospital death, treatment withdrawn, tracheotomy and condition aggravation were competing risk factors of weaning time, ICU duration or length of hospital stay, survival analysis and competing risk model were used to estimate and adjust the regression coefficients. Possibly post hoc analysis including per-protocol and subgroup analysis will be performed. Statistical analysis was performed using SPSS 23.0 (IBM, Armonk, New York) and R 3.6.1 software by “cmprsk” package.

**References**

1. Sessler CN, Wilhelm W, (2008) Analgesia and sedation in the intensive care unit: an overview of the issues. Crit Care 12: S1
2. Devlin JW, Skrobik Y, Gélinas C, Needham DM, Slooter AJC, Pandharipande PP, et al. Clinical Practice Guidelines for the Prevention and Management of Pain, Agitation/Sedation, Delirium, Immobility, and Sleep Disruption in Adult Patients in the ICU. Crit Care Med. 2018 Sep; 46(9): e825-e873.
3. Gommers D, Bakker J. Medications for analgesia and sedation in the intensive care unit: an overview. Crit Care. 2008; 12 Suppl 3: S4.
4. Soliman HM, Mélot C, Vincent JL: Sedative and analgesic practice in the intensive care unit: the results of a European survey. Br J Anaesth 2001, 87: 186-192.
5. Wunsch H, Kahn JM, Kramer AA, Rubenfeld GD: Use of intravenous infusion sedation among mechanically ventilated patients in the United States. Crit Care Med 2009, 37: 3031-3039.
6. Aragón RE, Proaño A, Mongilardi N, de Ferrari A, Herrera P, Roldan R, Paz E, Jaymez AA, Chirinos E, Portugal J, Quispe R, Brower RG, Checkley W. Sedation practices and clinical outcomes in mechanically ventilated patients in a prospective multicenter cohort. Crit Care. 2019 Apr 17; 23(1):130.8.
7. Hall RI, Sandham D, Cardinal P, Tweeddale M, Moher D, Wang X, Anis AH, Study Investigators. Propofol vs midazolam for ICU sedation, a Canadian multicenter randomized trial. CHEST 2001, 119: 1151-1159.
8. Carrasco G, Molina R, Costa J, Soler JM, Cabré L. Propofol vs midazolam in short-, medium-, and long-term sedation of critically ill patients, a cost–benefit analysis. Chest 1993, 103: 557-564.
9. Bauer TM, Ritz R, Haberthür C, Ha HR, Hunkeler W, Sleight AJ, Scollo-Lavizzari G, Haefeli WE. Prolonged sedation due to accumulation of conjugated metabolites of midazolam. Lancet 1995, 346: 145-147.
10. Barrientos-Vega R, Mar Sánchez-Soria M, Morales-García C, Robas-Gómez A, Cuena-Boy R, Ayensa-Rincon A. Prolonged sedation of critically ill patients with midazolam or propofol: impact on weaning and costs. Crit Care Med 1997, 25: 33-40.
11. Weinbroum AA, Halpern P, Rudick V, Sorkine P, Freedman M, Geller E: Midazolam versus propofol for long-term sedation in the ICU: a randomized prospective comparison. Intensive Care Med 1997, 23: 1258-1263.
12. Nimmo GR, Mackenzie SJ, Grant IS: Haemodynamic and oxygen transport effects of propofol infusion in critically ill adults. Anaesthesia 1994, 4: 485-489.
13. [Krajčová A](https://www.ncbi.nlm.nih.gov/pubmed/?term=Kraj%C4%8Dov%C3%A1%20A%5BAuthor%5D&cauthor=true&cauthor_uid=26558513), [Waldauf P](https://www.ncbi.nlm.nih.gov/pubmed/?term=Waldauf%20P%5BAuthor%5D&cauthor=true&cauthor_uid=26558513), [Anděl M](https://www.ncbi.nlm.nih.gov/pubmed/?term=And%C4%9Bl%20M%5BAuthor%5D&cauthor=true&cauthor_uid=26558513), [Duška F](https://www.ncbi.nlm.nih.gov/pubmed/?term=Du%C5%A1ka%20F%5BAuthor%5D&cauthor=true&cauthor_uid=26558513). Propofol infusion syndrome: a structured review of experimental studies and 153 published case reports. [Crit Care.](https://www.ncbi.nlm.nih.gov/pubmed/26558513) 2015 Nov 12; 19:398.
14. Gerlach AT, Dasta JF. Dexmedetomidine: An updated review. Ann Pharmacother. 2007; 41:245–52.
15. Hall JE, Uhrich TD, Barney JA, Arain SR, Ebert TJ. Sedative, amnestic, and analgesic properties of small dose dexmedetomidine infusions. Anesth Analg. 2000; 90:699–705.
16. Venn M, Newman J, Grounds M. A phase II study to evaluate the efficacy of dexmedetomidine for sedation in the medical intensive care unit. Intensive Care Med. 2003 Feb; 29(2):201-7.
17. Ruokonen E, Parviainen I, Jakob SM, Nunes S, Kaukonen M, Shepherd ST, Sarapohja T, Bratty JR, Takala J; "Dexmedetomidine for Continuous Sedation" Investigators. Dexmedetomidine versus propofol/midazolam for long-term sedation during mechanical ventilation. Intensive Care Med. 2009 Feb; 35(2):282-90.
18. Riker RR, Shehabi Y, Bokesch PM, Ceraso D, Wisemandle W, Koura F, Whitten P, Margolis BD, Byrne DW, Ely EW, Rocha MG; SEDCOM (Safety and Efficacy of Dexmedetomidine Compared With Midazolam) Study Group. Dexmedetomidine vs midazolam for sedation of critically ill patients: a randomized trial. JAMA. 2009 Feb 4; 301(5):489-99.
19. Jakob SM, Ruokonen E, Grounds RM, Sarapohja T, Garratt C, Pocock SJ, Bratty JR, Takala J; Dexmedetomidine for Long-Term Sedation Investigators. Dexmedetomidine vs midazolam or propofol for sedation during prolonged mechanical ventilation: two randomized controlled trials. JAMA. 2012 Mar 21; 307(11):1151-60.
20. [Shikha Gupta](https://www.ncbi.nlm.nih.gov/pubmed/?term=Gupta%20S%5BAuthor%5D&cauthor=true&cauthor_uid=25788780), [Dupinder Singh](https://www.ncbi.nlm.nih.gov/pubmed/?term=Singh%20D%5BAuthor%5D&cauthor=true&cauthor_uid=25788780), [Dinesh Sood](https://www.ncbi.nlm.nih.gov/pubmed/?term=Sood%20D%5BAuthor%5D&cauthor=true&cauthor_uid=25788780), and [Suneet Kathuria](https://www.ncbi.nlm.nih.gov/pubmed/?term=Kathuria%20S%5BAuthor%5D&cauthor=true&cauthor_uid=25788780). Role of dexmedetomidine in early extubation of the intensive care unit patients. [J Anaesthesiol Clin Pharmacol](https://www.ncbi.nlm.nih.gov/pmc/articles/PMC4353161/). 2015 Jan-Mar; 31(1): 92–98.
21. [Constantin JM](https://www.ncbi.nlm.nih.gov/pubmed/?term=Constantin%20JM%5BAuthor%5D&cauthor=true&cauthor_uid=26700947), [Momon A](https://www.ncbi.nlm.nih.gov/pubmed/?term=Momon%20A%5BAuthor%5D&cauthor=true&cauthor_uid=26700947), [Mantz J](https://www.ncbi.nlm.nih.gov/pubmed/?term=Mantz%20J%5BAuthor%5D&cauthor=true&cauthor_uid=26700947), [Payen JF](https://www.ncbi.nlm.nih.gov/pubmed/?term=Payen%20JF%5BAuthor%5D&cauthor=true&cauthor_uid=26700947), [De Jonghe B](https://www.ncbi.nlm.nih.gov/pubmed/?term=De%20Jonghe%20B%5BAuthor%5D&cauthor=true&cauthor_uid=26700947), [Perbet S](https://www.ncbi.nlm.nih.gov/pubmed/?term=Perbet%20S%5BAuthor%5D&cauthor=true&cauthor_uid=26700947), [Cayot S](https://www.ncbi.nlm.nih.gov/pubmed/?term=Cayot%20S%5BAuthor%5D&cauthor=true&cauthor_uid=26700947), [Chanques G](https://www.ncbi.nlm.nih.gov/pubmed/?term=Chanques%20G%5BAuthor%5D&cauthor=true&cauthor_uid=26700947), [Perreira B](https://www.ncbi.nlm.nih.gov/pubmed/?term=Perreira%20B%5BAuthor%5D&cauthor=true&cauthor_uid=26700947). Efficacy and safety of sedation with dexmedetomidine in critical care patients: a meta-analysis of randomized controlled trials. [Anaesth Crit Care Pain Med.](https://www.ncbi.nlm.nih.gov/pubmed/26700947) 2016 Feb; 35(1):7-15.
22. [Lachaine J](https://www.ncbi.nlm.nih.gov/pubmed/?term=Lachaine%20J%5BAuthor%5D&cauthor=true&cauthor_uid=22529402), [Beauchemin C](https://www.ncbi.nlm.nih.gov/pubmed/?term=Beauchemin%20C%5BAuthor%5D&cauthor=true&cauthor_uid=22529402). Economic evaluation of dexmedetomidine relative to midazolam for sedation in the intensive care unit. [Can J Hosp Pharm.](https://www.ncbi.nlm.nih.gov/pubmed/?term=Economic+Evaluation+of+Dexmedetomidine+Relative+to+Midazolam+for+Sedation+in+the+Intensive+Care+Unit) 2012 Mar; 65(2):103-10.
23. Pratik P Pandharipande, Brenda T Pun, Daniel L Herr, Mervyn Maze, Timothy D Girard, Russell R Miller, Ayumi K Shintani, Jennifer L Thompson, James C Jackson, Stephen A Deppen, Renee A Stiles, Robert S Dittus, Gordon R Bernard, E Wesley Ely. Effect of sedation with dexmedetomidine vs lorazepam on acute brain dysfunction in mechanically ventilated patients: the MENDS randomized controlled trial. JAMA. 2007 Dec 12; 298(22):2644-53.
24. Keating GM. Dexmedetomidine: A Review of Its Use for Sedation in the Intensive Care Setting. Drugs. 2015 Jul; 75(10):1119-30.
25. [Yongfang Zhou](https://www.ncbi.nlm.nih.gov/pubmed/?term=Zhou%20Y%5BAuthor%5D&cauthor=true&cauthor_uid=24935517), [Xiaodong Jin](https://www.ncbi.nlm.nih.gov/pubmed/?term=Jin%20X%5BAuthor%5D&cauthor=true&cauthor_uid=24935517), [Yan Kang](https://www.ncbi.nlm.nih.gov/pubmed/?term=Kang%20Y%5BAuthor%5D&cauthor=true&cauthor_uid=24935517), [Guopeng Liang](https://www.ncbi.nlm.nih.gov/pubmed/?term=Liang%20G%5BAuthor%5D&cauthor=true&cauthor_uid=24935517),[Tingting Liu](https://www.ncbi.nlm.nih.gov/pubmed/?term=Liu%20T%5BAuthor%5D&cauthor=true&cauthor_uid=24935517), and [Ni Deng](https://www.ncbi.nlm.nih.gov/pubmed/?term=Deng%20N%5BAuthor%5D&cauthor=true&cauthor_uid=24935517). Midazolam and propofol used alone or sequentially for long-term sedation in critically ill, mechanically ventilated patients: a prospective, randomized study. [Crit Care](https://www.ncbi.nlm.nih.gov/pmc/articles/PMC4095601/). 2014; 18(3): R122.
26. Gélinas C, Fillion L, Puntillo KA, et al. Validation of the critical-care pain observation tool in adult patients. Am J Crit Care 2006; 15:420-7.
27. Girard TD, Kress JP, Fuchs BD, Thomason JW, Schweickert WD, Pun BT, Taichman DB, Dunn JG, Pohlman AS, Kinniry PA, Jackson JC, Canonico AE, Light RW, Shintani AK, Thompson JL, Gordon SM, Hall JB, Dittus RS, Bernard GR, Ely EW. Efficacy and safety of a paired sedation and ventilator weaning protocol for mechanically ventilated patients in intensive care (Awakening and Breathing Controlled trial): a randomised controlled trial. Lancet. 2008 Jan 12; 371(9607):126-34.
28. Boles JM, Bion J, Connors A, Herridge M, Marsh B, Melot C, Pearl R, Silverman H, Stanchina M, Vieillard-Baron A, Welte T: Weaning from mechanical ventilation. Eur Respir J 2007, 29: 1033-1056.
29. SesslerCN, GosnellMS, GrapMJ, *et al*. The Richmond Agitation-Sedation Scale: validity and reliability in adult intensive care unit patients. Am J Respir Crit Care Med. 2002; 166(10):1338-1344.
30. ElyEW, InouyeSK, BernardGR, etal. Deliriumin mechanically ventilated patients: validity and reliability of the Confusion Assessment Method for the Intensive Care Unit (CAM-ICU). JAMA. 2001; 286 (21):2703-2710.
