# Supplementary material for: Sequential use of midazolam and dexmedetomidine for long-term sedation may reduce weaning time in selected critically ill, mechanically ventilated patients: a randomized controlled study
Source: Crit Care. 2022 May 3;26:122. doi: 10.1186/s13054-022-03967-5 (PMC9066885; doi:10.1186/s13054-022-03967-5)
Supplement: Supplementary file 2 — Additional file 2. Statistical analysis method and data supplement related to sedation, delirium, study outcomes by the per-protocol analysis and subgroups analysis, sedation-related costs, heart rate, and blood pressure. [file 13054_2022_3967_MOESM2_ESM.docx]

**Sequential Use of Midazolam and Dexmedetomidine for Long-Term Sedation May Reduce Weaning Time in Selected Critically Ill, Mechanically Ventilated Patients: A Randomized Controlled Study**

**Yongfang Zhou ^1^, M.M. Jie Yang ^1^, M.M. Bo Wang ^1^, PhD. Peng Wang ^1^, B.S. Zhen Wang ^1^, B.S. Yunqing Yang ^1^, B.S. Guopeng Liang ^1^, M.M. Xiaorong jing ^1^, B.S. Xiaodong Jin ^1^, MD. Zhongwei Zhang ^1^, MD. Yiyun Deng ^1^, PhD. Chenggong Hu ^1^, PhD. Xuelian Liao ^1^, PhD. Wanhong Yin ^1^, PhD. Zhihong Tang ^1^. MS. Yongming Tian ^1^, M.M. Liyuan Tao ^2^, PhD. Yan Kang ^1*^, MD.**

**Author affiliations:  ^1^ Department of Critical Care Medicine, West China Hospital of Sichuan University, Chengdu, Sichuan 610041, China. ^2^Research Center of Clinical Epidemiology, Peking University Third Hospital, Beijing 100191, China.**

**^*^ Correspondence author: Yan Kang, MD**

**Address: Guoxue Alley 37#, Wuhou District,**

**Chengdu, Sichuan Province, 610041.**

**Email:** [**kangyan@scu.edu.cn**](mailto:kangyan@scu.edu.cn)**.**

**Additional file method and results**

**Contents**

**Additional file S1. Sample Size Calculation..............................................Section.1**

**Additional file S2. Statistical Analysis......................................................Section.2**

**Additional file S3. Results.....................................................................Section.3**

eFigure 1**...................................................................3.1**

eFigure 2**...................................................................3.2**

eFigure 3**...................................................................3.3**

eFigure 4**...................................................................3.4**

Table S1**....................................................................3.5**

Table S2**....................................................................3.6**

Table S3**....................................................................3.7**

Table S4**....................................................................3.8**

Table S5**....................................................................3.9**

Table S6**....................................................................3.10**

**S1. Sample Size Calculation**

The planned enrollment was 252 participants in this trial. The previous study showed that means (±standard deviations) of weaning time for midazolam, propofol and dexmedetomidine were 97.9 ± 54.6h, 34.8 ± 29.4h and 24.2 ± 1.67h in long-term sedation respectively ^11, 25^. Considering there are huge differences in weaning time among these medications and fairly minor difference between propofol and dexmedetomidine, we assumed weaning time to be 34.8 hours in group M-P and it would be reduced by 12 hours in group M-D with clinical significance, and the standard deviation (25.2h) was calculated by combining variances of propofol and dexmedetomidine. A sample size of 213 of three groups was thus estimated to give 80% power and a two-sided significance level of 0.05. As some patients possibly withdrew the treatment, 252 patients were enrolled for the study in order to manage a 15% dropout rate.

**S2. Statistical Analysis**

Data were primarily analyzed following the intention-to-treatment principle (ITT). Post hoc analysis including per-treatment and subgroup analysis were also performed. Values of normal distribution were expressed by mean ± standard deviation, values of non-normal distribution were expressed as median and interquartile range (IQR) and categorical variables as counts and percentages. For continuous variables analysis, the differences in normally distributed data of the three groups were compared with one-way analysis of variance and differences between any two groups were analyzed by Student–Newman–Keuls (SNK) methods, P<0.05 was considered statistically significant. For all other variables, the significance level was 0.05 for comparisons of three groups, with adjustment to 0.017 for comparisons for any two groups. Differences in non-normally distributed variables among the three groups were assessed using Kruskal–Wallis analysis of variance and differences between any two groups were compared with Mann–Whitney 𝑈 test. Categorical variables were analyzed using Chi-squared test or Fisher's exact test. Considering death on ventilator, treatment withdrawn, and tracheotomy were competing risk factors of weaning time; in-ICU death and treatment withdrawn were competing risk factors of length of ICU stay; and in-hospital death and treatment withdrawn were competing risk factors of length of hospital stay, survival analysis and competing risk model analysis were used to assess the effect of the treatment protocols on weaning time, length of ICU stay and hospital stay. The treatment protocols (group M-D, group M-P, or group M) were introduced as two dummy variables to obtain hazard ratios, odds ratios, or mean difference for comparison with the reference group. The mean (95%CIs) of between-group differences of the median were calculated by the bootstrap method (10000 replications). The 95%CI of absolute rate differences between groups were calculated using the Newcombe-Wilson score method. Statistical significance was defined as P<0.05 with 2-sided testing. Statistical analysis was performed using SPSS 23.0 and R project.

**S3. Results**

**3.1 eFigure 1**

**
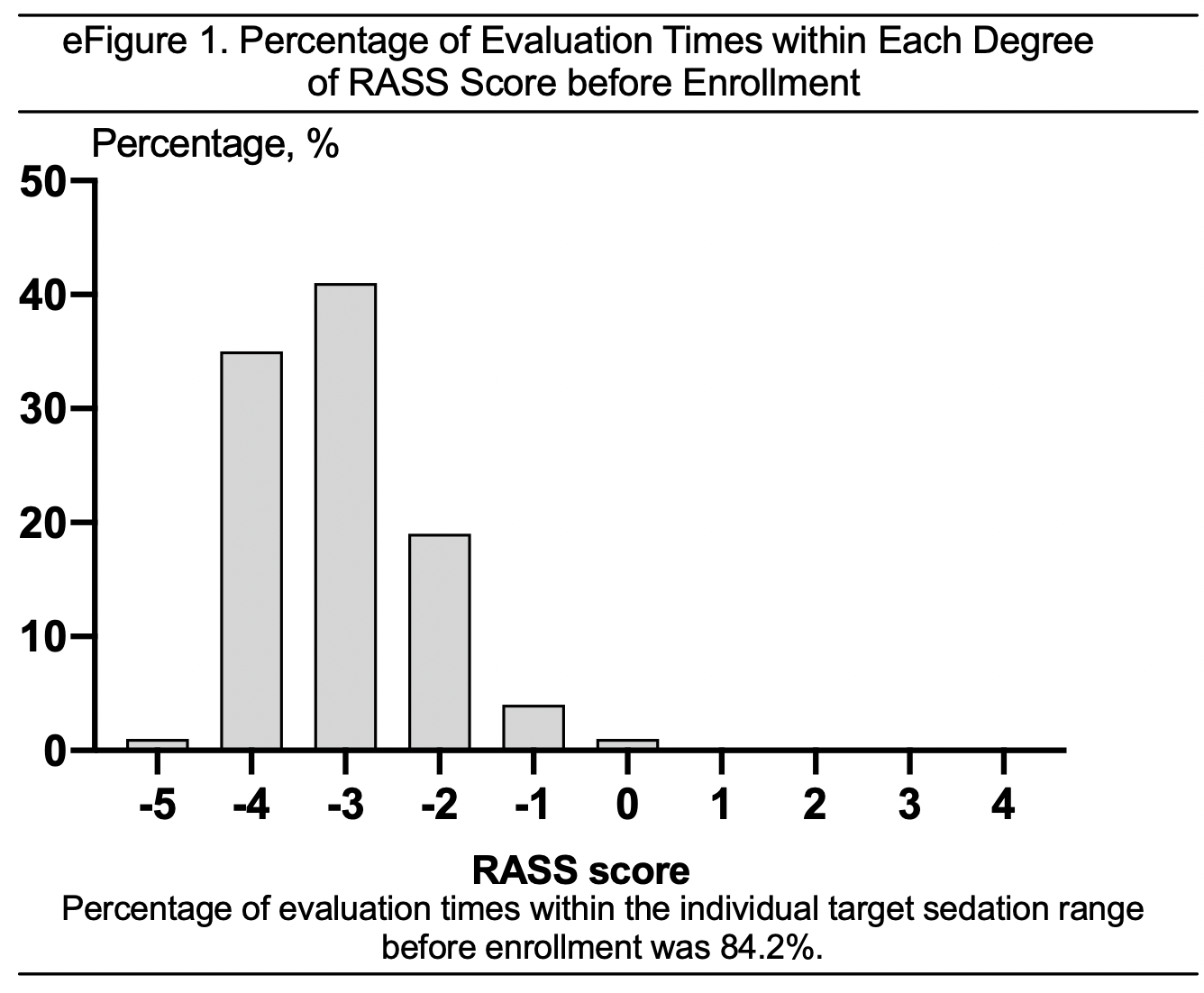
**

**3.2 eFigure 2**

**
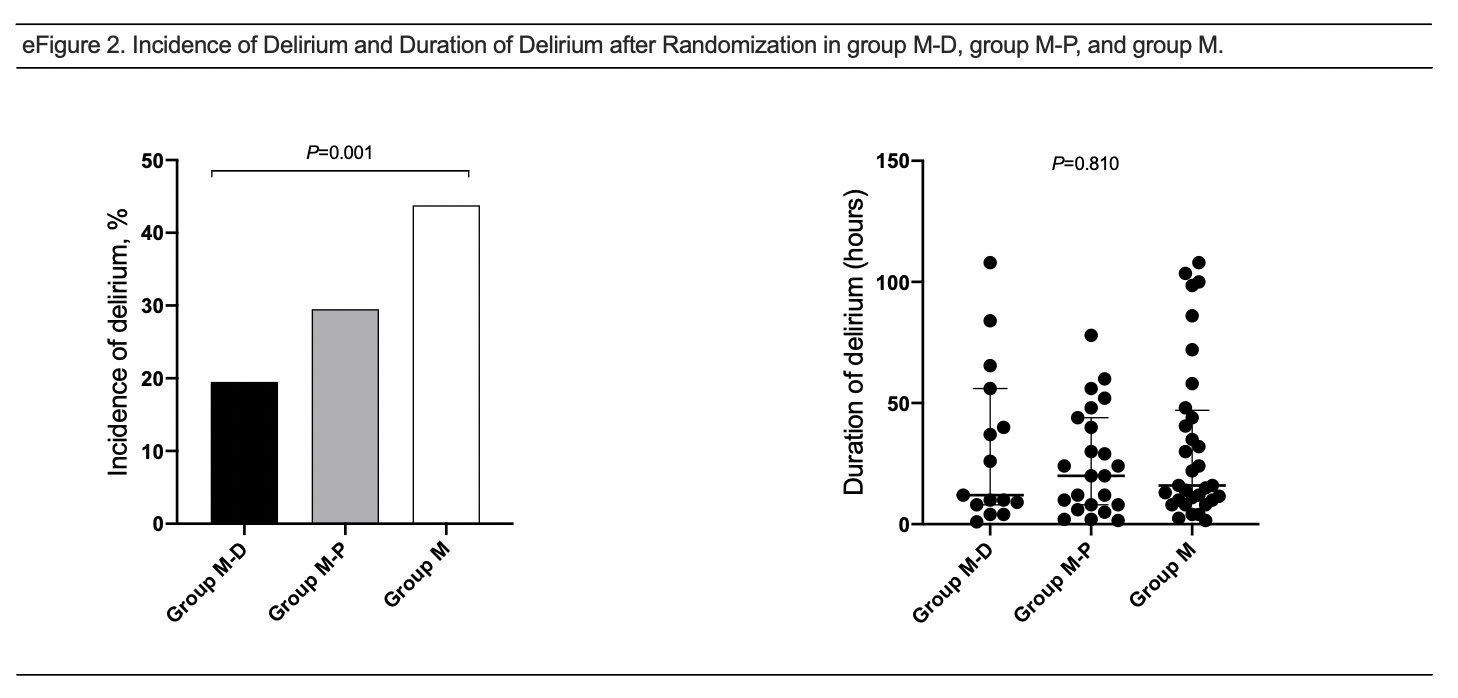
**

**3.3 eFigure 3**


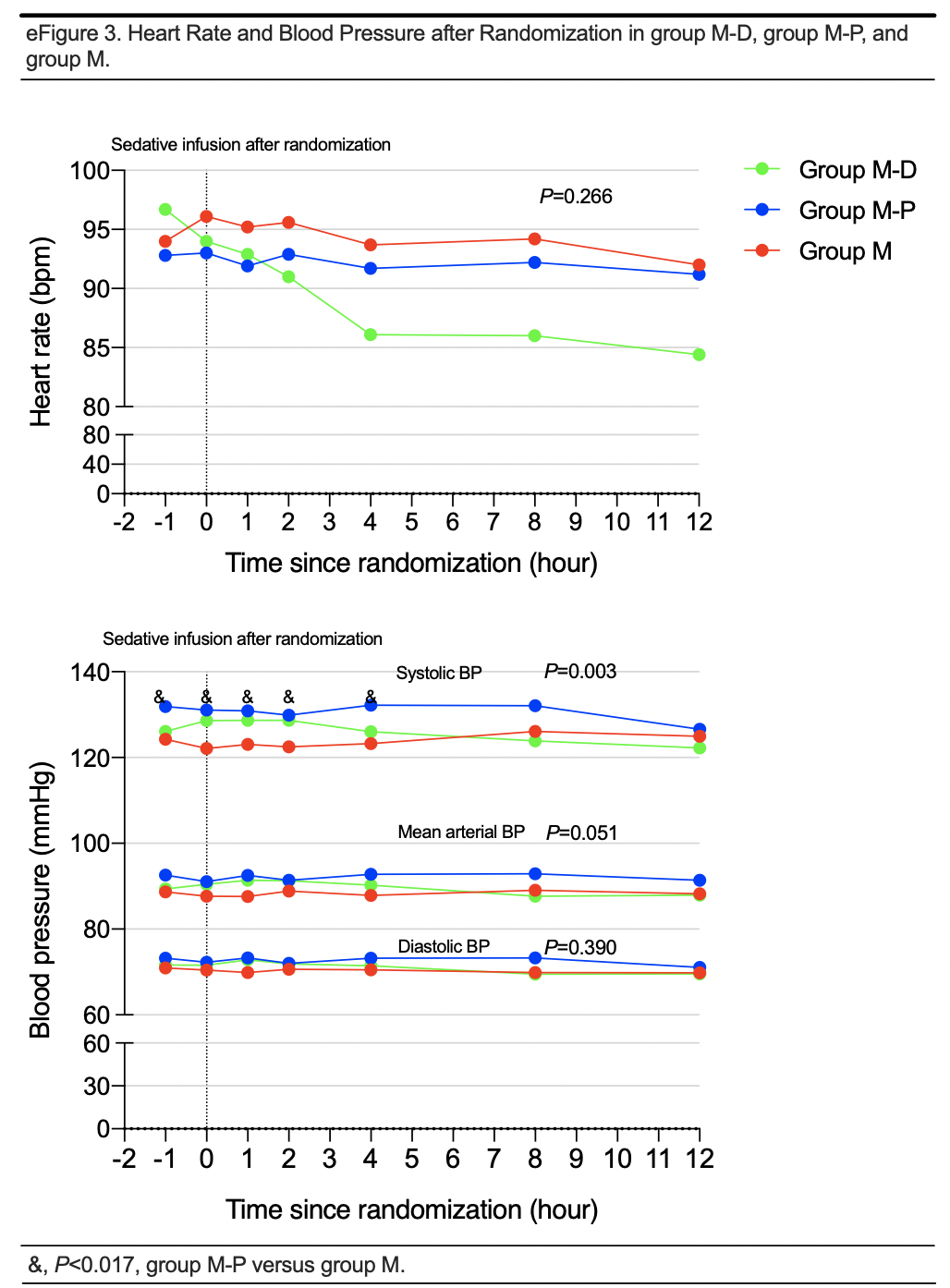


**eFigure 4**

**
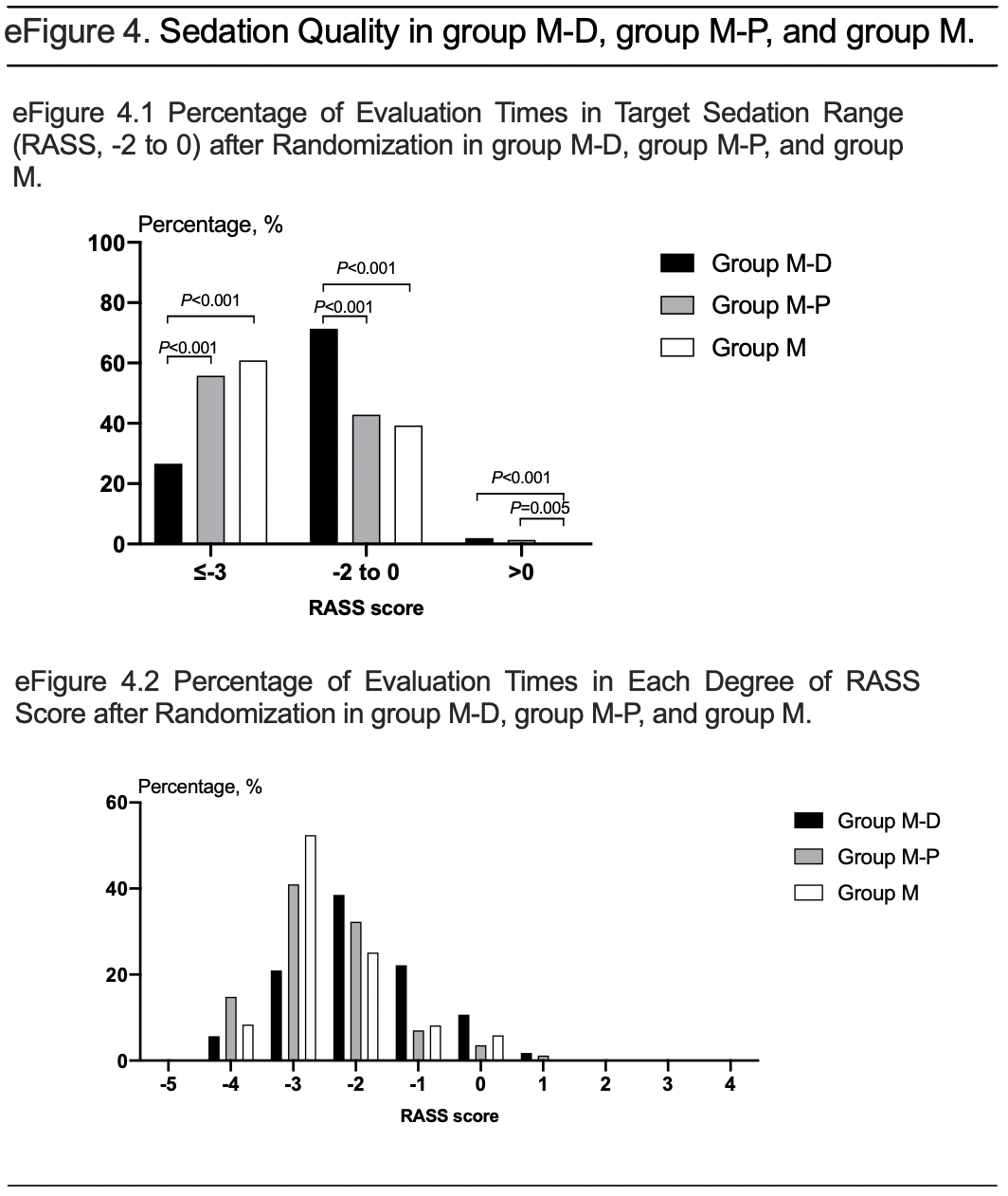
**

**3.5 Table S1**

| **Table S1. Study Outcomes by the Per-Protocol Analysis ^a^** | | | | | | | |
| --- | --- | --- | --- | --- | --- | --- | --- |
|  |  |  |  |  | **Value of Pairwise comparison** | | |
|  |  |  |  |  | **1 vs 2** | **1 vs 3** | **2 vs 3** |
| **Outcome Measure** | **1 Group M-D (n=70)** | **2 Group M-P (n=73)** | **3 Group M (n=65)** | ***P* value** | ***P* value** | ***P* value** | ***P* value** |
| Weaning time ^b^, median (IQR), h | 25.0 (17.5-48.0) | 31.3 (23.0-50.0) | 49.0 (30.5-72.2) | <0.001 | 0.039 | <0.001 | 0.051 |
| Recovery time, median (IQR), h | 0.4 (0.0-1.5) | 2.0 (1.0-4.5) | 7.0 (2.0-28.0) | <0.001 | <0.001 | <0.001 | <0.001 |
| Extubation time, median (IQR), h | 0.8 (0.0-7.5) | 3.5 (1.5-5.5) | 8.0 (2.2-30.2) | <0.001 | 0.002 | <0.001 | 0.002 |
| Sequential sedation time, median (IQR), h | 24.0 (13.0-35.5) | 25.3 (20.0-47.5) | 25.0 (24.0-45.0) | 0.018 | 0.028 | 0.008 | 0.603 |
| Delirium, No. (%) | 15 (21.4) | 23 (31.5) | 30 (46.2) | 0.009 | 0.173 | 0.002 | 0.077 |
| The dose of fentanyl after randomization, median (IQR), mg | 1.5 (1.0-2.5) | 2.5 (1.0-4.0) | 2.0 (1.2-3.5) | 0.020 | 0.015 | 0.016 | 0.967 |
| ICU duration ^b^, median (IQR), d | 14.8 (9.9-18.5) | 14.1 (9.8-19.2) | 17.9 (10.5-23.8) | 0.040 | 0.143 | 0.006 | 0.397 |
| Length of hospital stay ^b^, median (IQR), d | 19.3(13.4-25.7) | 19.9(15.0-35.9) | 22.9(16.6-40.3) | 0.051 | NA | NA | NA |
| Adverse effects, No. (%) | 5 (7.1) | 12 (16.4) | 7 (10.8) | 0.214 | NA | NA | NA |
| Hypotension, No. (%) | 4 (5.7) | 2 (2.7) | 0 (0.0) | 0.169 | NA | NA | NA |
| Bradycardia, No. (%) | 1 (1.4) | 1 (1.4) | 0 (0.0) | 1.000 | NA | NA | NA |
| Hypertension, No. (%) | 0 (0.0) | 8 (11.0) | 5 (7.7) | 0.010 | 0.006 | 0.024 | 0.512 |
| Tachycardia, No. (%) | 1 (1.4) | 1 (1.4) | 2 (3.1) | 0.691 | NA | NA | NA |
| Triglyceride, median (IQR), mmol/L | 1.76 (1.20-2.21) | 1.81 (1.28-2.37) | 1.58 (1.19-2.46) | 0.891 | NA | NA | NA |
| Abbreviations: IQR, Interquartile Range; ICU, Intensive Care Unit; NA, not applicable.  ^a^ Calculation was based on 208 patients completing study protocol and successful extubation, excluding 8 patients in group M (1 death, 1 withdrawing treatment, 1 tracheotomy, and 5 extubation failure), 5 patients in group M-P (1 withdrawing treatment, 1 tracheotomy, 1 condition aggravation, and 2 extubation failure), and 7 patients in group M-D (1 death, 2 withdrawing treatment, 1 tracheotomy, 2 condition aggravation, and 1 extubation failure).  ^b^ Calculated using competing risk model analysis.  Recovery time: the time from sedation cessation to awakening.  Extubation time: the time from sedation cessation to extubation.  Weaning time: the time from randomization to extubation.  Sequential sedation time: the sedation period after randomization.  ICU duration: the time from admission to ICU until discharge from ICU.  Length of hospital stay: the time from patient screened until discharge from hospital.  SI conversion factors: To convert cholesterol to Triglyceride to milligrams per deciliter, multiply by 88.50. | | | | | | | |

**3.6 Table S2**

| **Table S2. Study Outcomes in the Subgroup of Mechanical Ventilation Duration** < **5 Days before Randomization** | | | | | | | |
| --- | --- | --- | --- | --- | --- | --- | --- |
|  |  |  |  |  | **Value of Pairwise Comparison** | | |
|  |  |  |  |  | **1 vs 2** | **1 vs 3** | **2 vs 3** |
| **Outcome Measure** | **1 Group M-D (n=34)** | **2 Group M-P (n=37)** | **3 Group M (n=34)** | ***P* value** | ***P* value** | ***P* value** | ***P* value** |
| Weaning time^a^, median (IQR), h | 25.1 (17.5-48.0) | 30.5 (21.5-48.5) | 48.8(26.3-72.2) | 0.087 | NA | NA | NA |
| Recovery time ^b^，median(IQR), h | 0.5 (0.0-1.8) | 2.0 (1.0-4.8) | 7.8 (1.8-30.2) | <0.001 | 0.004 | <0.001 | 0.032 |
| Extubation time ^b^, median (IQR), h | 4.5 (0.0-9.0) | 4.0 (1.0-5.0) | 8.0 (2.0-48.0) | 0.018 | 0.805 | 0.011 | 0.019 |
| Sequential sedation time ^b^, median (IQR), h | 19.0 (11.0-35.0) | 25.0 (20.1-38.8) | 24.4 (23.5-30.0) | 0.073 | NA | NA | NA |
| Successful extubation, No. (%) | 33 (97.1) | 35 (94.6) | 33 (97.1) | 1.000 | NA | NA | NA |
| Tracheotomy, No. (%) | 0 (0.0) | 1 (2.7) | 0 (0.0) | 1.000 | NA | NA | NA |
| Delirium, No. (%) | 8 (23.5) | 13 (35.1) | 13 (38.2) | 0.391 | NA | NA | NA |
| The dose of fentanyl after randomization, median (IQR), mg | 1.5 (0.9-2.0) | 2.0 (1.0-3.5) | 1.5 (1.2-2.4) | 0.065 | NA | NA | NA |
| ICU duration ^a^, median (IQR), d | 10.3(7.9-14.9) | 9.9 (7.9-14.1) | 10.6 (7.6-17.9) | 0.433 | NA | NA | NA |
| Length of hospital stay ^a^, median (IQR), d | 15.7 (10.8-20.7) | 18.9 (11.5-21.6) | 18.1 (11.4-20.8) | 0.175 | NA | NA | NA |
| ICU mortality, No. (%) | 0 (0.0) | 2 (5.4) | 3 (8.8) | 0.277 | NA | NA | NA |
| Hospital mortality, No. (%) | 0 (0.0) | 2 (5.4) | 3 (8.8) | 0.277 | NA | NA | NA |
| Adverse effects, No. (%) | 3 (8.8) | 4 (10.8) | 2 (5.9) | 0.907 | NA | NA | NA |
| Hypotension No. (%) | 3 (8.8) | 1 (2.7) | 0 (0.0) | 0.211 | NA | NA | NA |
| Bradycardia No. (%) | 1 (2.9) | 1 (2.7) | 0 (0.0) | 1.000 | NA | NA | NA |
| Hypertension, No. (%) | 0 (0.0) | 2 (5.4) | 2 (5.9) | 0.544 | NA | NA | NA |
| Tachycardia, No. (%) | 0 (0.0) | 0 (0.0) | 0 (0.0) | 1.000 | NA | NA | NA |
| Triglyceride, median (IQR), mmol/L | 1.65 (1.13-2.00) | 1.49 (1.22-2.36) | 1.46 (1.12-2.44) | 0.963 | NA | NA | NA |
| Abbreviations: IQR, Interquartile Range; ICU, Intensive Care Unit; NA, not applicable.  ^a^ Calculated using competing risk model analysis.  ^b^ Calculation was based on 102 patients completing study protocol, excluding 0 patient in group M, 2 patients in group M-P (1 withdrawing treatment and 1 tracheotomy), and 1 patient in group M-D (1 condition aggravation).  Recovery time: Time from stopping sedation to awakening.  Extubation time: Time from stopping sedation to extubation.  Weaning time: Time from randomization to extubation.  Sequential sedation time: Sedation administration period after randomization.  ICU duration: Time from patient admission to ICU until discharge from ICU.  Length of hospital stay: Time from patient screened until discharge from hospital.  SI conversion factors: To convert cholesterol to Triglyceride to milligrams per deciliter, multiply by 88.50. | | | | | | | |

**3.7 Table S3**

| **Table S3. Study Outcomes in the Subgroup of Mechanical Ventilation Duration** ≥ **5 Days before Randomization** | | | | | | | |
| --- | --- | --- | --- | --- | --- | --- | --- |
|  |  |  |  |  | **Value of Pairwise Comparison** | | |
|  |  |  |  |  | **1 vs 2** | **1 vs 3** | **2 vs 3** |
| **Outcome Measure** | **1 Group M-D (n=43)** | **2 Group M-P (n=41)** | **3 Group M (n=39)** | ***P* value** | ***P* value** | ***P* value** | ***P* value** |
| Weaning time ^a^, median (IQR), hour | 25.0 (20.1-46.5) | 41.5 (23.5-52.0) | 49.0 (27.0-73.3) | 0.137 | NA | NA | NA |
| Recovery time ^b^，median(IQR), hour | 0.1 (0.0-1.0) | 2.0 (1.5-4.0) | 6.3 (2.5-25.0) | <0.001 | <0.001 | <0.001 | 0.005 |
| Extubation time ^b^, median (IQR), hour | 0.3 (0.0-1.5) | 3.0 (1.6-6.3) | 7.0 (2.5-25.0) | <0.001 | <0.001 | <0.001 | 0.040 |
| Sequential sedation time ^b^, median (IQR), hour | 24.1 (18.3-47.0) | 31.8 (19.8-48.3) | 25.7 (24.0-47.5) | 0.191 | NA | NA | NA |
| Successful extubation, No. (%) | 37 (86.0) | 38 (92.7) | 32 (82.1) | 0.359 | NA | NA | NA |
| Tracheotomy, No. (%) | 2 (4.7) | 0 (0.0) | 4 (10.3) | 0.081 | NA | NA | NA |
| Delirium, No. (%) | 7 (16.3) | 10 (24.4) | 19 (48.7) | 0.004 | 0.355 | 0.002 | 0.024 |
| The accumulated dose of fentanyl after randomization (mg) | 1.5 (1.0-3.1) | 3.0 (1.0-4.0) | 3.0 (1.0-5.8) | 0.063 | NA | NA | NA |
| ICU duration ^a^, median (IQR), days | 16.8 (11.7-23.2) | 17.0 (14.0-21.8) | 24.9 (19.0-32.9) | 0.195 | NA | NA | NA |
| Length of hospital stay ^a^, median (IQR), days | 21.9 (15.0-26.9) | 21.8 (15.3-36.6) | 33.7 (24.7-44.6) | 0.572 | NA | NA | NA |
| ICU mortality, No. (%) | 2 (4.7) | 5 (12.2) | 2 (5.1) | 0.463 | NA | NA | NA |
| Hospital mortality, No. (%) | 2 (4.7) | 5 (12.2) | 2 (5.1) | 0.463 | NA | NA | NA |
| Adverse effects, No. (%) | 2 (4.7) | 9 (22.0) | 5 (12.8) | 0.062 | NA | NA | NA |
| Hypotension, No. (%) | 1 (2.3) | 1 (2.4) | 0 (0.0) | 1.000 | NA | NA | NA |
| Bradycardia, No. (%) | 0 (0.0) | 0 (0.0) | 0 (0.0) | 1.000 | NA | NA | NA |
| Hypertension, No. (%) | 0 (0.0) | 7 (17.1) | 3 (7.7) | 0.009 | 0.005 | 0.103 | 0.205 |
| Tachycardia, No. (%) | 1 (2.3) | 2 (4.9) | 2 (5.1) | 0.739 | NA | NA | NA |
| Triglyceride, median (IQR), mmol/L | 1.79 (1.21-2.55) | 2.03 (1.57-2.37) | 1.67 (1.11-2.60) | 0.796 | NA | NA | NA |
| Abbreviations: IQR, Interquartile Range; ICU, Intensive Care Unit; NA, not applicable.  ^a^ Calculated using competing risk model analysis.  ^b^ Calculation was based on 114 patients completing study protocol, excluding 3 patients in group M (1 death, 1 withdrawing treatment, and 1 tracheotomy), 1 patient in group M-P (1 condition aggravation), and 5 patients in group M-D (1 death, 2 withdrawing treatment, 1 tracheotomy, and 1 condition aggravation).  Recovery time: Time from stopping sedation to awakening.  Extubation time: Time from stopping sedation to extubation.  Weaning time: Time from randomization to extubation.  Sequential sedation time: Sedation administration period after randomization.  ICU duration: Time from patient admission to ICU until discharge from ICU.  Length of hospital stay: Time from patient screened until discharge from hospital.  SI conversion factors: To convert cholesterol to Triglyceride to milligrams per deciliter, multiply by 88.50. | | | | | | | |

**3.8 Table S4**

| **Table S4. Economical outcomes analyzed by intention-to-treatment analysis** | | | | | | | |
| --- | --- | --- | --- | --- | --- | --- | --- |
|  |  |  |  |  | **Value of Pairwise Comparison** | | |
|  |  |  |  |  | **1 vs 2** | **1 vs 3** | **2 vs 3** |
| **Outcome Measure** | **1 Group M-D (n=77)** | **2 Group M-P (n=78)** | **3 Group M (n=73)** | ***P* value** | ***P* value** | ***P* value** | ***P* value** |
| The acquisition cost of sedatives after randomization, median (IQR), yuan | 273.1 (136.6, 546.2) | 723.0 (482.0, 1254.0) | 327.3 (163.7, 625.0) | <0.001 | <0.001 | 0.291 | <0.001 |
| The total acquisition cost of sedatives, median (IQR), yuan | 1364.9 (688.5, 2242.5) | 2143.0 (1243.0, 3330.0) | 1375.2 (650.0, 2922.9) | 0.001 | <0.001 | 0.374 | 0.010 |
| The total cost of ICU treatment, median (IQR), yuan | 86305.4 (57405.2, 131459.8) | 95575.8 (48489.6, 131320.3) | 108726.1 (63332.7, 188196.0) | 0.082 | NA | NA | NA |

Abbreviation: IQR, interquartile range.

**3.9 Table S5**

| **Table S5. Heart Rate after Randomization in group M-D, group M-P, and group M** | | | | | | | |
| --- | --- | --- | --- | --- | --- | --- | --- |
|  | Baseline data | Time post sedative infusion after randomization | | | | | |
|  | 1h before randomization | 0.5h | 1h | 2h | 4h | 8h | 12h |
| Group M-D | 96.7±18.9 | 94.0±18.5 | 92.9±18.9 | 91.0±18.5 | 86.1±19.0# | 86.0±18.3# | 84.4±17.0# |
| Group M-P | 92.8±19.5 | 93.0±19.5 | 91.9±20.1 | 92.9±19.4 | 91.7±19.0 | 92.2±19.4 | 91.2±17.9 |
| Group M | 94.0±17.9 | 96.1±17.7 | 95.2±17.4 | 95.6±17.2 | 93.7±18.5 | 94.2±17.9 | 92.0±18.5 |

#p<0.05 versus baseline

| **Table S6. Blood Pressure after Randomization in group M-D, group M-P, and group M** | | | | | | | |
| --- | --- | --- | --- | --- | --- | --- | --- |
| Mean arteria blood pressure | Baseline data | Time post sedative infusion after randomization | | | | | |
|  | 1h before randomization | 0.5h | 1h | 2h | 4h | 8h | 12h |
| Group M-D | 90.3±12.9 | 90.4±12.4 | 91.4±13.1 | 91.3±12.1 | 90.3±12.1 | 87.7±14.8 | 87.9±14.2 |
| Group M-P | 92.6±13.3 | 91.6±12.0 | 92.5±11.9 | 91.4±13.5 | 92.8±13.2 | 92.9±14.5 | 91.4±14.1 |
| Group M | 88.7±13.2 | 87.7±12.0 | 87.6±11.1 | 88.9±12.4 | 87.9±12.2 | 89.1±12.5 | 88.2±13.5 |
| Systolic blood pressure |  |  |  |  |  |  |  |
| Group M-D | 126.1±16.2 | 128.6±18.6 | 128.7±19.3 | 128.7±17.1 | 126.6±16.3 | 123.9±18.0 | 122.3±18.4 |
| Group M-P | 131.9±18.6& | 131.1±18.1& | 130.9±16.1& | 129.9±19.7& | 132.2±18.1& | 132.1±19.5 | 130.6±18.5 |
| Group M | 124.3±18.1 | 122.1±15.8 | 123.1±15.6 | 122.5±19.6 | 123.3±17.4 | 126.1±17.9 | 124.9±17.0 |
| Diastolic blood pressure |  |  |  |  |  |  |  |
| Group M-D | 71.6±12.3 | 71.6±11.8 | 72.8±12.4 | 71.9±13.0 | 71.4±12.3 | 69.5±11.4 | 69.6±12.3 |
| Group M-P | 73.2±14.1 | 72.3±12.8 | 73.3±12.3 | 72.0±13.6 | 73.2±13.7 | 73.3±14.0 | 71.1±15.5 |
| Group M | 70.9±13.3 | 70.4±12.8 | 69.9±11.6 | 70.6±15.0 | 70.5±11.8 | 69.9±14.5 | 69.8±13.7 |

**3.10 Table S6**

&p<0.017 versus group M
